# Down-regulation of ARNT promotes cancer metastasis by activating the fibronectin/integrin β1/FAK axis

**DOI:** 10.18632/oncotarget.3448

**Published:** 2015-03-20

**Authors:** Chi-Ruei Huang, Chung-Ta Lee, Kwang-Yu Chang, Wen-Chang Chang, Yao-Wen Liu, Jenq-Chang Lee, Ben-Kuen Chen

**Affiliations:** ^1^ Institute of Bioinformatics and Biosignal Transduction, College of Bioscience and Biotechnology, National Cheng Kung University, Taiwan, ROC; ^2^ Graduate Institute of Medical Sciences, College of Medicine, Taipei Medical University, Taiwan, ROC; ^3^ Department of Pathology, National Cheng Kung University Hospital, Taiwan, ROC; ^4^ National Institute of Cancer Research, National Health Research Institutes, Taiwan; ^5^ Division of Hematology/Oncology, Department of Internal Medicine; National Cheng Kung University Hospital, College of Medicine, National Cheng Kung University, Taiwan; ^6^ Department of Pathology, Kuo General Hospital, Taiwan, ROC; ^7^ Department of Surgery, National Cheng Kung University Hospital, Taiwan, ROC; ^8^ Department of Pharmacology, College of Medicine, National Cheng Kung University, Taiwan, ROC; ^9^ Institute for Cancer Biology and Drug Discovery, College of Medical Science and Technology, Taipei Medical University, Taiwan, ROC

**Keywords:** Metastasis, ARNT, Oncogene, ROS

## Abstract

The aryl hydrocarbon receptor nuclear translocator (ARNT) is broadly involved in regulating tumorigenesis by inducing genes that are involved in tumor growth and angiogenesis. Tumorigenesis usually involves normoxic conditions. However, the role of ARNT in tumor metastasis during normoxia remains unclear. Here, we demonstrate that ARNT protein levels were decreased in late-stage human colorectal cancer using immunohistochemical analysis. Down-regulation of ARNT protein promoted cancer cell migration and invasion, which was mediated by activation of the fibronectin/integrin β1/FAK signaling axis. In addition, the enhancement of migration and invasion in ANRT knockdown cells was blocked when ARNT was restored in the cells. In xenografts in severe combined immunodeficiency mice, tumor growth was significantly inhibited in the ARNT-knockdown condition. However, the tail-vein injection animal model revealed that the depletion of ARNT-induced metastatic lung colonies was further enhanced when ARNT expression was recovered post-injection. Interestingly, chemotherapeutic drugs inhibited ARNT expression and promoted the invasion of residual tumor cells. These results suggest that ARNT may play a positive role during tumor growth (either in early-stage tumor growth or in organ metastases), but plays a negative role in tumor migration and invasion. Therefore, the efficiency of ARNT-targeted therapy during different cancer stages should be carefully evaluated.

## INTRODUCTION

The aryl hydrocarbon receptor (AHR) nuclear translocator (ARNT) (also known as hypoxia-inducible factor (HIF)-1β) is a member of the basic helix-loop-helix PER/AHR/ARNT/SIM (bHLH-PAS) family of transcription factors [1]. ARNT serves as a dimerization partner for a number of transcription factors, such as the AHR and c-Jun proteins, and mediates the regulation of TCDD-responsive genes, cyclooxygenase (COX)-2, 12(*S*)-lipoxygenase, p21*^WAF1/CIP1^* and multidrug resistance 1 (MDR1) under normoxic conditions, thereby contributing to tumorigenesis and drug resistance [1–5]. In addition, ARNT forms a heterodimer with HIF-1α in response to varying oxygen levels within microenvironments and promotes cell survival and angiogenesis [6, 7]. Loss of HIF-1α and ARNT also leads to an increased response to radiotherapy, a reduction in tumor growth, and decreased angiogenesis in tumors transplanted into immune-deficient mice [8]. These studies indicate that ARNT interacts with specific transcription factors in response to environmental conditions to trigger the signaling of tumorigenesis under either normoxic or hypoxic conditions.

ARNT expression has been documented in several cancers. It is interesting to note that *ARNT* is located at chromosome 1q21.3, a region that is amplified in several cancers with *MCL-1* gene amplification and inhibits cancer cell apoptosis [9]. An ARNT/ETV6 hybrid transcript has been described in two cases of leukemia due to t(1;12)(q21;p13) translocation [10, 11]. In addition, ARNT splice variants promote the progression of estrogen receptor-negative breast cancer [12]. ARNT is required for tumor initiation in tumors induced by environmental toxicities such as benzo[α]pyrene exposure [13]. Therefore, the expression of ARNT in tumors appears to be a prognostic biomarker and a target for cancer therapies.

Cancer lethality is a result of metastasis—the process in which cancer cells transfer from their original location to distal organs—and metastatic cancer cells are more malignant and resistant to anticancer drugs [14]. The success of metastasis is regulated by a process called epithelial-mesenchymal transition (EMT). EMT has also been shown to occur during the initiation of metastasis as cancer progresses [15]. Several protein markers are known to mediate cancer mobility during the EMT process. For example, fibronectin, which is a component of the extracellular matrix (ECM) and a ligand for integrins outside cells, participates in wound healing and embryonic development [16]. By activating its downstream integrin β1/FAK signaling pathway, fibronectin promotes cell adhesion and migration [17]. Integrin β1 is a transmembrane receptor that mediates the attachment between tumor cells and their surroundings, such as stromal cells or the extracellular matrix. Fibronectin couples with other α-type integrins to transmit extracellular signals and activate FAK [18].

ARNT expression is required for tumor cell growth in most cancers [19, 20]; therefore, ARNT is considered a target for cancer therapy. However, its role during metastasis has not been investigated under normoxic conditions. To characterize the potential function of ARNT in the regulation of metastasis under normoxic conditions, the effect of ARNT on tumor metastasis was evaluated in cancer cell lines and human cancer tissues. Our study demonstrates that the loss of ARNT induces a cascade of events, which results in a pro-metastatic phenotype in colorectal cancer. We found that ARNT depletion directly upregulated the fibronectin/integrin β1/FAK signaling axis, which promoted EMT and metastasis. Furthermore, ARNT expression was inversely correlated with cancer stage in human colorectal cancer.

## MATERIALS AND METHODS

### Cell lines and reagents

The cell line of human melanoma cells (A375) was grown at 37°C under 5% CO2 in 10 cm plastic dishes containing 10 ml of Dulbecco's modified Eagle's medium supplemented with 10% fetal bovine serum, 100 μg/ml streptomycin, and 100 units/ml penicillin. The cell line of human colon adenocarcinoma, SW480 was also grown in same manner but with different media, including Leibovitz's L-15 Medium. The cell lines of C4 and vT2 were also grown in same manner but with Minimum Essential Media (MEM) and 0.1 mg/ml G418 for vT2 cells. In order to stable silenced ARNT, cells was infected with lentivirus-based shRNA clone and selected with puromycin (Sigma Corporation, Cream Ridge, NJ, USA). In some experiments, Tet-inducible vector which was express ARNT^KDR^-Myc was transfected in shARNT cells and selected with hygromycin B (AppliChem Gmbh, Darmstadt, Hessen, Germany) in doxycycline (Sigma Corporation, Cream Ridge, NJ, USA) containing DMEM for a concentration of 1 μg/ml.

### Plasmid construction

ARNT^KDR^ cDNA fragments were generated by QuikChange Site-Directed Mutagenesis Kit (Stratagene, Inc., La Jolla, CA, USA) from pcDNA3.1 Myc/His ARNT plasmid [21]. ARNT 3′UTR and 3′UTR mut cDNA fragments were generated by RT-PCR and subcloned into PmeI site of pcDNA3.1 Myc/His ARNT plasmid and XbaI site of pGL3 promoter according to previous report [22]. Hypoxia response element (HRE)-Luciferase reporter was constructed by primer assembling using PCR machine and subcloned into BglII and HindIII site of pGL3 according to previous report [23]. The construct of miR-107 inhibitor was purchased from GeneCopoeia, Inc. (GeneCopoeia, Inc., Rockville, MD, USA). DNA fragment of miR-107 was generated by PCR from genomic DNA and subcloned into pEZX-AM02. All clones were confirmed by DNA sequencing.

### Transfection of cells with plasmids and luciferase assay

Transient transfection of cells with plasmids was performed with Lipofectamine 2000 (Invitrogen, Life technologies, Carlsbad, CA, USA) according to the manufacturer's instructions but with slight modifications. Cells were replated 24 h before transfection at a density of 3 × 10^5^ cells in 2 ml of fresh culture medium in a 3.5-cm plastic dish. For use in transfection, 2 μl of Lipofectamine 2000 was incubated with 1 μg of indicated plasmid at room temperature. Cells were incubated at 37°C in a humidified atmosphere of 5% CO2 before harvested. The luciferase activity in cell lysate was determined as described previously [24].

### shRNA clones and lentivirus infection

shRNA clones were obtained from the National RNAi Core Facility Platform located at the Institute of Molecular Biology / Genomic Research Center, Academia Sinica, supported by the National Core Facility Program for Biotechnology Grants of NSC (NSC 100–2319-B-001–002). Individual clones should be identified by their unique TRC number (e.g. shARNT: TRCN0000003819, shLacZ: TRCN0000072223, shFibronectin: TRCN0000064828, shITGB1: TRCN0000029648, shFAK: TRCN0000121129, shAhR: TRCN0000245286). Lentivirus infection was following standard protocol with minor modify. Briefly, 1 × 10^5^ cells was seeding to each well in 6-well plates for overnight. Before lentivirus was infection, the culture media was changed to the fresh media containing 8 μg/mL polybrene (Sigma Corporation, Cream Ridge, NJ, USA). Lentivirus was added to the cells at MOI = 3. Following additional incubation of infected cells overnight, infected cells was selected with 1 μg/mL puromycin.

### Immunofluorescence

Cells grown on chamber slides and fixed with 4% paraformaldehyde (Sigma Corporation, Cream Ridge, NJ, USA) in phosphate-buffered saline at 25°C for 30 min. The cells were then rinsed with phosphate-buffered saline three times and permeabilized, blocked with 1% Triton X-100, 2% FBS for 15 min. Next, the cells were incubated with indicated polyclonal antibodies at a dilution of 1:100 for 1 h and treated with Alexa Fluor^®^ 546 goat anti-rabbit IgG polyclonal antibodies at a dilution of 1:500 or combine with Alexa Fluor^®^ 488 Phalloidin at a dilution of 1:200 (Invitrogen, Life technologies, Carlsbad, CA, USA) for 30 min. Finally, the cells were counterstained with 0.2 μg/μl 4, 6-diamidino-2-phenylindole (Invitrogen, Life technologies, Carlsbad, CA, USA) and mounted in 90% glycerol mounting solution. Digital picture was examined by using a microscope Olympus BX51 (Olympus America, Inc., Melville, NY, USA).

### Reverse transcription-PCR

Total RNA was isolated using the TRIzol^®^ RNA isolation reagents (Invitrogen, Life technologies, Carlsbad, CA, USA), and 1 μg of RNA was subjected to reverse transcription-PCR with GoScript™ Reverse Transcription system (Promega Corporation, Madison, Wisconsin, USA). The *fibronectin*-specific primers (sense, 5′-GCAGCCACAACTTCTCTGG TCCTC-3′; antisense, 5′-AACAACCGGGCTTGCTT TGACT-3′), and *GAPDH*-specific primers (sense, 5′-CCATCACCATCTTCCAGGAG-3′; antisense, 5′-CCTGCTTCACCACCTTCTTG-3′) were used. The PCR products were separated by 2% agarose gel electrophoresis and visualized with ethidium bromide staining.

### Western blotting

An analytical 10% sodium dodecyl sulfate poly acrylamide gel electrophoresis (SDS-PAGE) was performed, and 30 μg of protein were analyzed, unless stated otherwise. For immuno-blotting, proteins in the SDS gels were transferred onto a polyvinylidene difluoride membrane by an electroblot apparatus. Antibodies against human ARNT (Cell Signaling Technology, Inc., Danvers, MA, USA), α-Tubulin (Sigma Corporation, Cream Ridge, NJ, USA), N-cadherin, β-catenin, E-cadherin Vimentin, Fibronectin, Integrin β1, and phospho-FAK^Y397^ (all from Epitomics, Inc., Burlingame, CA, USA) were used as the primary antibodies. Mouse or rabbit IgG antibodies coupled to horseradish peroxidase were used as secondary antibodies. An enhanced chemiluminescence kit (Pierce, Rockford, IL) was used for detection.

### Wound healing assay

For wound healing assay, 3.5 × 10^4^ cells were seeding in 12-wells containing the linear spacer inserts. Following overnight culture, the linear spacer inserts was removed, which created a regular and defined “wound” within the cell monolayer. After phosphate-buffered saline wash, wells were either left untreated or treated with FAK inhibitor 14 (1, 2, 4, 5-Tetraaminobenzene tetrahydrochloride) (Sigma Corporation, Cream Ridge, NJ, USA) in 0.5% FBS medium. The extent of wound closure was observed by using a phase-contrast microscope camera Olympus BX51 (Olympus America, Inc., Melville, NY, USA).

### Transwell migration assay

Cells were trypsinized and 3.5 × 10^4^ cells were added to the Boyden chambers (8 μm pore size; Millipore, Billerica, MA, USA), in 0.5% FBS containing medium, and assay media with 10% FBS was added to the culture plates. After incubation for 15 h, the nonmotile cells at the top of the filter were removed and the motile cells at the bottom of the filter were fixed with 4% paraformaldehyde and stained with a one-tenth dilution of Giemsa (Sigma Corporation, Cream Ridge, NJ, USA). The number of migrating cells in each chamber was counted in five randomly chosen fields under the microscope for three independent experiments.

### Transwell invasion assay

Cells were plated in serum-free medium on the upper Boyden chamber coated with 100 μl of 10% matrigel, with serum-containing medium in the lower chamber. Two days later, cells on the apical side of each insert were scraped off, and invading cells on the basolateral side of the membrane were fixed and stained as the same as transwell migration assay. The number of invading cells was counted in three randomly chosen fields under the microscope for three independent experiments.

### Transendothelial invasion assay

HMEC1 cells (1 × 10^5^ cells per well) were plated on the upper chamber and allowed to grow to confluence, and then loaded 100 μl of 10% matrigel into the chamber. Tumor cells were stained with 1, 1′-dioctadecyl-3, 3, 3′, 3′-tetramethyl-indocarbocyanine perchlorate (DiI) (Invitrogen, Life technologies, Carlsbad, CA, USA) for 30 min. DiI stained 1.5 × 10^5^ tumor cells were then loaded into the chamber and incubated for 2 days. Cells on the apical side of each insert were scraped off. Invasion to the basolateral side of the membrane was visualized with the immunofluorescent microscope. The number of invading cells was counted in three randomly chosen fields under the microscope for three independent experiments.

### Patients and materials

We randomly recruited 38 colorectal cancers of various tumor-node-metastasis (TNM) stages [25]. (2 stage 0, 5 stage I, 6 stage II, 13 stage III, and 12 stage IV cancers) from patients who had undergone surgery for primary colorectal cancer between 2008 and 2009 at National Cheng Kung University Hospital. The status of the tumors was recorded according to the TNM staging system. The stage of the tumors was determined from the histopathologic reports obtained at the time of resection. Patients who had received neoadjuvant chemotherapy or radiotherapy before their initial resection were excluded from this study.

### Tumor metastasis assay in animal model

Tumor metastasis was determined by tail vein intravenous injection of cancer cells into 4- to 6-week-old male severe combined immunodeficiency (SCID) mice. Briefly, each animal was injected with 1 × 10^6^ cells mixed with phosphate-buffered saline, and all mice were sacrificed until 2 month after injection. All mice were obtained from the National Cheng Kung University Laboratory Animal Center (Tainan, Taiwan) and the National Laboratory Animal Center (Tainan, Taiwan). All animal experiments in this study were approved by the Laboratory Animal Committee of National Cheng Kung University. H&E stained were performed by the Human Biobank, Research Center of Clinical Medicine, National Cheng Kung University Hospital. In tet-regulated ARNT expression experiment, mice were pre-feed with doxycycline (1 μg/ml) containing water. Following 3 days after 1 × 10^6^ cells was injected into tail vein of mice, the mice were divided into two groups randomly. One group of mice was maintained in doxycycline containing water continuously, the other was replaced with clean water. The body weight was measured every 2–3 days. After 6 weeks injection, mice were sacrificed. Lung tissues were harvested and embedded with paraffin. Hematoxylin and eosin stain were performed to visual the cancer cells metastasis.

### Immunohistochemistry

Tissue sections were obtained from a representative paraformaldehyde-fixed paraffin-embedded tissue block of each patient's tumor. Tissue blocks containing a transmural, full-thickness section of adenocarcinoma, including the deepest pericolonic extension, were selected. Immunohistochemistry (IHC) was performed essentially as previously described [26]. Briefly, endogenous peroxidase activity of tissue sections was blocked with H_2_O_2_. Slides were incubated in boiling citrate buffer (pH 6.0), then maintained at a sub-boiling temperature for 10 min. Cool slides on bench top for 30 min. The sections were incubated at 4°C for overnight with anti-ARNT antibodies (diluted 1:100) (Cell Signaling Technology, Inc., Danvers, MA, USA). The reaction complexes were detected using a kit (VECTASTAIN Elite ABC kits) and visualized using a 3, 3-diaminobenzidine (DAB) substrate kit (Vector DAB substrate). Finally, the sections were lightly counterstained with hematoxylin. All IHCs were assessed by a pathologist with 10 years' experience (C-T Lee). The nuclear positivity of ARNT was counted. The IHC results of ARNT were grouped into “no expression” (no IHC reactivity or < 5% of positive cells/total tumor cells), “low expression” (faint or light brown nuclear staining in at least 5% of total tumor cells), and “high expression” (dark brown nuclear staining in at least 5% of total tumor cells).

### Statistical analysis

Statistical analysis was performed with either *t*-tests (for comparison between two groups), one-way ANOVA analysis of variance (Tukey's or Newman-Keuls' post-tests, for comparison among multiple experimental groups) or two-way ANOVA analysis of variance (for compare the effects of different drink water (with or without doxycycline 1 μg/ml) at different time intervals) using GraphPad Prism 4.0 software (GraphPad Software, San Diego, CA, USA). A Fisher's exact test and Kendall's tau (τ)-b correlation analysis were used to examine the relationship between the expression levels of ARNT and various clinicopathologic features. Data are shown as mean ± s.e.m. of three independent experiments or are counted at least fifteen microscopic fields per condition. A *P*-value less than 0.05 was considered significant and was denoted by *. The *P*-value less than 0.01 and 0.001 was denoted by ** and ***, respectively. n.s.: no significant difference.

## RESULTS

### The depletion of ARNT promotes cancer cell migration and invasion

**Figure 1:**
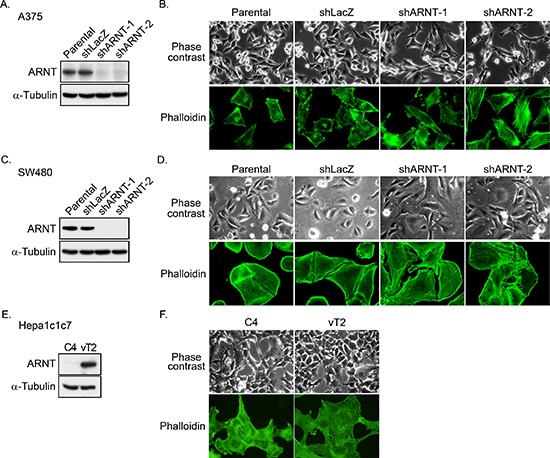
ARNT silencing promotes morphological changes in tumor cells **(A)** Lentivirus-based shRNA against *ARNT* was infected into A375 cells. Cell lysates were prepared and subjected to SDS-PAGE and then analyzed by Western blotting with antibodies against ARNT and α-Tubulin. **(B)** Parental and shARNT cells were fixed using 4% paraformaldehyde and labeled with F-actin-specific fluorescently labeled phalloidin. Immunofluorescence images were acquired using a microscope. **(C)** Lentivirus-based shRNA against *ARNT* was infected into SW480 cells. Cell lysates were prepared and subjected to SDS-PAGE and then analyzed by Western blotting with antibodies against ARNT and α-Tubulin. **(D)** Cells were fixed using 4% paraformaldehyde and labeled with F-actin-specific fluorescently labeled phalloidin. Immunofluorescence images were acquired using a microscope. **(E)** Expression of ARNT and α-Tubulin in C4 and vT2 cells was examined using Western blotting with antibodies against ARNT and α-Tubulin, respectively. **(F)** Cells were fixed using 4% paraformaldehyde and labeled with F-actin-specific fluorescently labeled phalloidin. Immunofluorescence images were acquired using a microscope.

ARNT is constitutively expressed in a wide range of tissues, and its expression is correlated with tumorigenesis [8]. Although tumorigenesis usually involves normoxic conditions, the functional role of ARNT in the regulation of tumor metastasis is not well characterized under normoxic conditions. To examine whether ARNT participates in tumor metastasis in normoxia, stable cell lines in which ARNT was knocked down via short hairpin (sh)RNA (shARNT) were confirmed and used (Figures 1A and 1C). The hypoxia-induced promoter contains hypoxia response element (HRE) binding sites (Supplementary Figure S1), and its activity was inhibited in shARNT cells, indicating the loss of ARNT function in shARNT cells. Interestingly, shARNT cells had increased spindle-shaped morphology and cytoskeletal rearrangements (Figures 1B and 1D). To confirm whether the changes in cellular morphology were accompanied by the depletion of ARNT, we examined the hepatoma C4 cell line, in which ARNT is degraded by the replacement of Gly326 with asparagine, and vT2 cells, which were derived from C4 cells possessing complete transfection of ARNT cDNA [27] (Figure 1E). Consistent with the results observed in the shARNT cell lines, the spindle-shaped morphology in C4 cells was diminished in ARNT-expressing vT2 cells (Figure 1F), suggesting that ARNT depletion may promote cell mobility.

**Figure 2:**
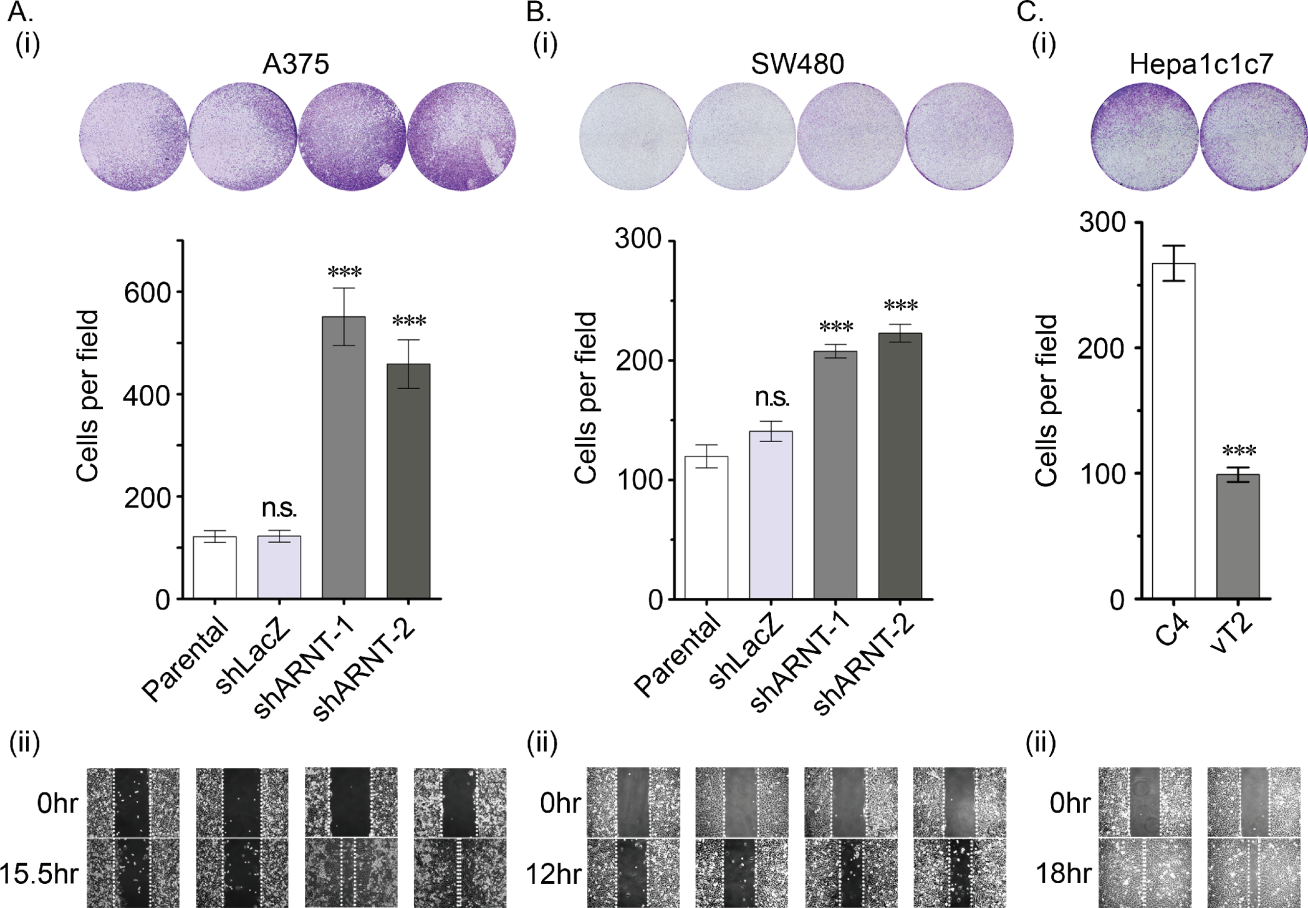
Loss of ARNT increases tumor cell migration (A–C) The migration properties of A375 (A) SW480 **(B)** and Hepa1c1c7 **(C)** cells were analyzed by transwell migration (i) and wound-healing (ii) assays as described in the “Materials and methods”. In the trans-well assay (i), migrating cells were examined using a microscope (upper panel). The number of migrating cells was counted in three randomly chosen fields from three independent experiments (lower panel). Values are indicated as the mean ± s.e.m. ***P < 0.001; n.s.: no significant difference.

**Figure 3:**
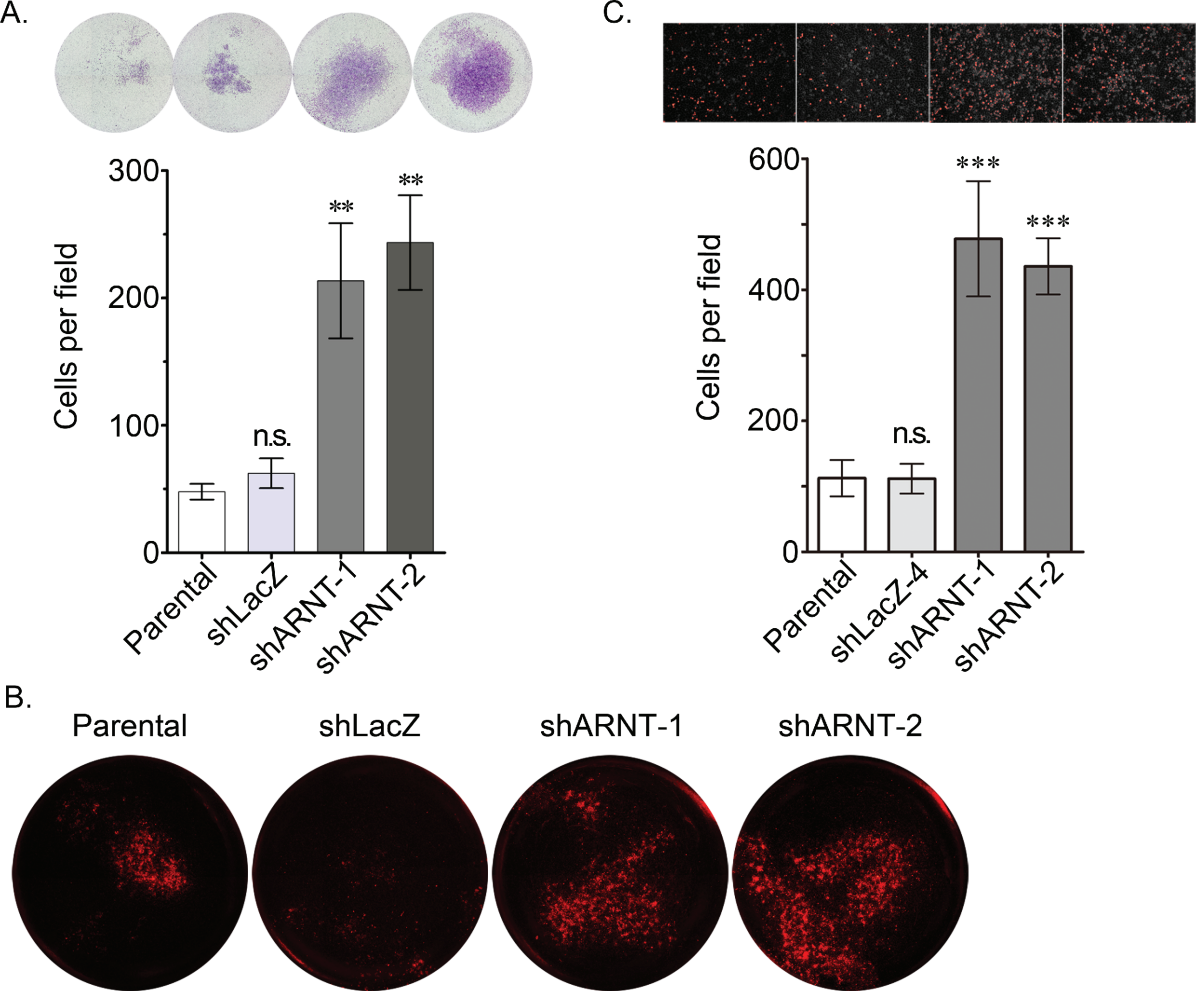
Loss of ARNT increases tumor cell invasion **(A)** The invasive properties of cancer cells were examined using an invasion assay as described in the “Materials and methods”. Images for the invasion assay were examined using a microscope (upper panel). The number of invading cells was counted using three randomly chosen fields from three independent experiments (lower panel). Values are indicated as the mean ± s.e.m. **(B)** The transendothelial invasion of cancer cells was performed as described in the “Materials and methods”. The invasive images were examined using a microscope. **(C)** Parental, shARNT and shLacZ cells were labeled with DiI and cultured with endothelial cells for 3 h. The attachment of cells was examined using a microscope (upper panel). The number of attached cells was counted using three randomly chosen fields from three independent experiments (lower panel). Values are indicated as the mean ± s.e.m. **: *P* < 0.01; ***: *P* < 0.001; n.s.: no significant difference.

To clarify whether the morphological changes observed in the shARNT cells affected cell mobility, cell migration was examined using trans-well and wound-healing assays. As shown in Figures 2A and 2B, cell migration was significantly increased in shARNT cells. In addition, compared with C4 cells, migration was repressed in ARNT-expressing vT2 cells (Figure 2C). The trans-well and transendothelial invasion assays were also used to investigate whether ARNT depletion conferred invasive properties to tumor cells. As shown in Figures 3A and 3B, a significant increase in the number of penetrated tumor cells was observed in the ARNT knockdown conditions. The interaction between tumor cells and endothelial cells was also enhanced in shARNT cells (Figure 3C), indicating that the adhesion of tumor cells to blood vessels may be negatively regulated by ARNT. Collectively, these results revealed that ARNT down-regulation promoted cancer cell migration and invasion.

### ARNT depletion promotes activation of the fibronectin/integrin β1/FAK signaling pathway

**Figure 4:**
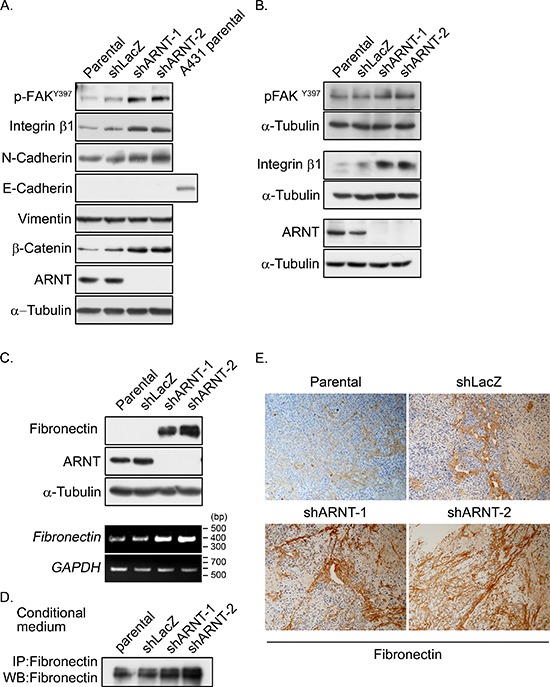
Expression of integrin β1 and fibronectin and activation of FAK are increased in ARNT knockdown cells **(A–B)** Cell lysates of A375 **(A)** and SW480 **(B)** cells were prepared and subjected to SDS-PAGE and then analyzed by Western blotting with antibodies against phosphorylated FAK^Y397^, integrin β1, N-cadherin, E-cadherin, Vimentin, β-catenin, ARNT and α-Tubulin. **(C)** Cell lysates were prepared and subjected to SDS-PAGE and then analyzed by Western blotting with antibodies against fibronectin, ARNT and α-Tubulin (upper panel). Total RNA was extracted for reverse transcription PCR with *fibronectin* and *glyceraldehyde-3-phosphate dehydrogenase* (*GAPDH*) primers (lower panel). **(D)** Secreted fibronectin was harvested from conditioned medium and detected with antibody-mediated immunoprecipitation. **(E)** Fibronectin protein was detected in xenografted SCID mice. Primary tumors were harvested, embedded in paraffin, sectioned and stained with either hematoxylin and antibodies against fibronectin.

Based on the observation that ARNT depletion enhanced cancer metastasis, we further investigated the mechanisms that were involved in the regulation of ARNT-mediated cell migration. The dissemination of cancer cells from primary tumors is the result of an EMT process [15]. Therefore, we examined the proteins that are involved in EMT. As shown in Figure 4A, the expression levels of E-cadherin and vimentin were not changed in shARNT cells. However, expression of N-cadherin was increased and co-localized with F-actin fibers in shARNT cells (Figure 4A and Supplementary Figure S2A). Subsequently, to investigate the possibility that FAK activation was essential for the increased cell motility, we examined FAK activation in shARNT cells. As shown in Figures 4A and 4B, ARNT depletion stimulated FAK phosphorylation (p-FAK^Y397^), which accumulated in the tips of filopodia (Supplementary Figure S2B). In addition, the effect of FAK activation on cell migration and invasion in shARNT cells was studied using FAK inhibitor 14. As shown in Supplementary Figure S3, FAK inhibitor 14 significantly reduced the amount of phospho-FAK in shARNT cells. As expected, cell migration and invasion were also abolished in shARNT cells treated with FAK inhibitor 14 (Supplementary Figure S4). These results indicate that ARNT attenuation triggered FAK activation, resulting in increased tumor migration and invasion.

In general, FAK activation is mediated by the activating ECM-integrin signaling pathway. Indeed, increased integrin β1, as well as increased phospho-FAK, were observed in shARNT cells (Figures 4A and 4B), indicating that integrin pathway activation may contribute to cell migration in shARNT cells. Therefore, we examined the expression level of fibronectin, which is a component of the ECM and an integrin ligand in ARNT knockdown cells. Interestingly, ARNT depletion significantly increased the expression of fibronectin (Figure 4C). Immunofluorescence staining also demonstrated increased fibronectin protein levels in ARNT-deficient cells (Supplementary Figure S5). As a ligand of integrin receptors, the secretion of fibronectin outside of cells is required for its cellular function. Therefore, we further studied whether ARNT depletion-induced secreted fibronectin was present in the culture media. As shown in Figure 4D, fibronectin secretion into the culture media was increased in shARNT cells. We also examined the expression and secretion of fibronectin in tumor tissues using the xenograft model. The expression and secretion of fibronectin was increased in mice injected with shARNT cells (Figure 4E). These results suggest that ARNT depletion induced fibronectin expression. We showed previously that reactive oxygen species (ROS) levels were increased in shARNT cells [4, 5]. Lin et al. also demonstrated that ROS promote fibronectin expression through ERK activation [28]. Therefore, we hypothesized that the increase of fibronectin expression, resulting in FAK activation, may be caused by the increase of ROS in shARNT cells. As shown in Supplementary Figures S6A and S6B, the ROS scavenger N-acetylcysteine significantly inhibited FAK phosphorylation and migration in shARNT cells. The induction of fibronectin expression in shARNT cells was also blocked by the ERK inhibitor U0126 (Supplementary Figure S6C). These results suggest that the elevated ROS levels in shARNT cells may activate ERK signaling to promote activation of the fibronectin/integrin β1/FAK axis.

**Figure 5:**
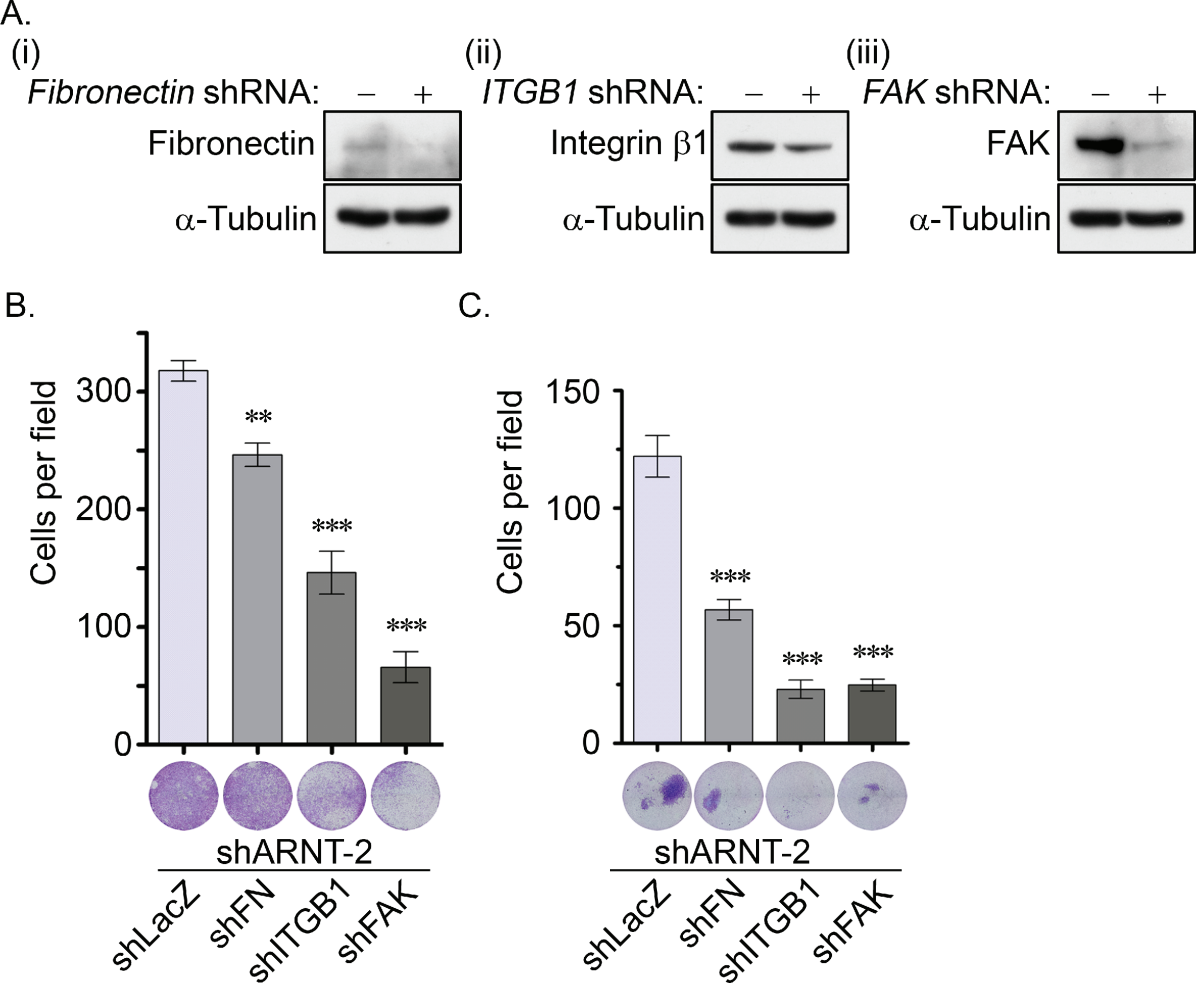
The fibronectin/integrin β1/FAK signaling pathway axis regulates ARNT knockdown-induced cell migration and invasion **(A)** Lentivirus-based shRNA against fibronectin (*FN*, i), integrin β1 (*ITGB1*, ii) and focal adhesion kinase (*FAK*, iii) were infected into cells. Cell lysates were prepared and subjected to SDS-PAGE and then analyzed by Western blotting with antibodies against fibronectin, integrin β1, FAK and α-Tubulin. **(B–C)** The migration and invasive properties of cancer cells were examined using migration **(B)** and invasion **(C)** assays as described in the “Materials and methods”. The images for the migration and invasion assay were examined using a microscope (lower panel). The number of migrating and invasive cells was counted using three randomly chosen fields from three independent experiments (upper panel). Values are indicated as the mean ± s.e.m. **: *P* < 0.01; ***: *P* < 0.001.

To characterize the role of the fibronectin/integrin β1/FAK axis in the motility changes of the ARNT knockdown cells, lentivirus-based shRNAs against *fibronectin*, *integrin β1* and *FAK* were applied to the cells to eliminate fibronectin, integrin β1 and FAK, respectively (Figure 5A). As shown in Figures 5B and 5C, the shARNT-induced cell migration and invasion were significantly inhibited in the fibronectin, integrin β1, and FAK knockdown conditions. These results demonstrate that the fibronectin/integrin β1/FAK axis was responsible for the ARNT depletion-induced tumor cell migration and invasion.

### ARNT recovery in shARNT cells is essential for metastatic tumor growth

**Figure 6:**
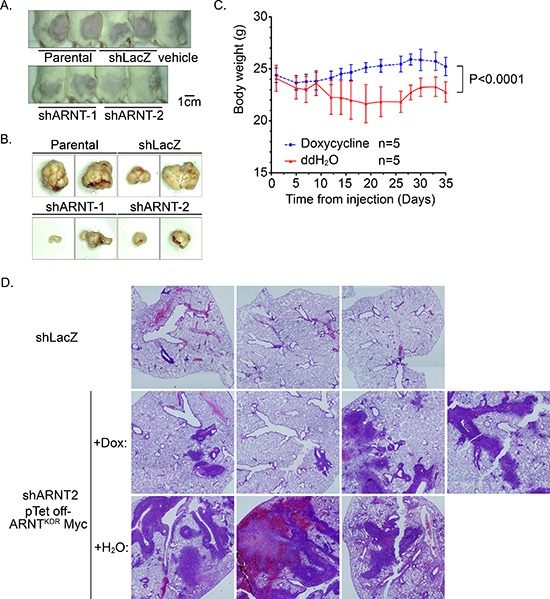
RNT recovery in shARNT cells is essential for xenograft and metastatic tumor growth **(A–B)** Cells (1 × 10^6^) were injected into the right leg of mice. Tumor growth was monitored externally using Vernier calipers for up to 4 weeks **(A)** The mice were sacrificed to harvest the tumors **(B) (C–D)** After shARNT, cells with pTet off-ARNT^KDR^ Myc were injected into the tail vein of the mice, and two groups were divided based on whether doxycycline was added to the water. Body weight was measured every 2–3 days **(C)** After 6 weeks, the mice were sacrificed and the lungs harvested for H & E staining **(D)** Images were taken on a microscope at 20X magnification and merged from multiple fields.

To the best of our knowledge, ARNT expression is an essential factor for promoting tumor growth. To confirm that ARNT is essential for tumor growth, an *in vivo* xenograft model was used. As shown in Figures 6A and 6B, tumor growth was reduced in shARNT cells. Furthermore, we analyzed *ARNT* gene copy number across multiple cancer types using the Tumorscape database (http://www.broadinstitute.org/tumorscape/pages/portalHome.jsf). Strikingly, similar to *MCL-1* [9], *ARNT* is located at chromosome 1q21.3 and was significantly amplified (Supplementary Figure S7A). The database search also revealed that *ARNT* was amplified in 36% of all the cancers analyzed (*q* value = 1.61 × 10^−49^) and, importantly, that it mapped to a peak of amplification present in almost 13% of colorectal cancers (Supplementary Figure S7B). We also analyzed the Cancer Cell Line Encyclopedia (http://www.broadinstitute.org/software/cprg/) to study *ARNT* gene-copy changes in human cancers [29]. As shown in Supplementary Figure S7C, *ARNT* was amplified in almost all cancer cell lines, and its amplification was correlated with *MCL-1* amplification (r^2^ = 0.9184) (Supplementary Figure S7D). These results indicate that ARNT expression is essential for tumor growth.

Our studies indicated that ARNT improved tumor growth but blocked tumor migration and invasion. Therefore, to further investigate if recovery of ARNT expression was required for the growth of shARNT cells following metastatic seeding of the lungs, a Tet-off inducible system that inhibited ARNT^KDR^-Myc expression was used. The expression and function of ARNT^KDR^-Myc, an ARNT mutant that is not targeted by shRNA and retains its function, was verified in shARNT cells under hypoxic conditions (Supplementary Figure S8A). As shown in Figure S8B, ARNT^KDR^-Myc was expressed in shARNT cells; however, treatment with doxycycline repressed ARNT^KDR^-Myc expression but increased phospho-FAK^Y397^ and fibronectin expression. The induction of ARNT^KDR^-Myc by doxychcline removal also repressed the migration and invasion of shARNT cells and their interaction with endothelial cells (Supplementary Figures S8C–S8E). These results suggest that FAK activation and fibronectin expression may enhance tumor metastasis, which was dependent on the level of ARNT controlled by the Tet-off inducible system in shARNT cells. In addition, we injected shARNT cells expressing doxycycline-regulated ARNT^KDR^-Myc into the tail vein of SCID mice that were fed doxycycline. Three days later, two groups of mice were separated into a doxycycline-removed group or a control group. Loss of body weight was observed in doxycycline-removed mice (Figure 6C), indicating that ARNT recovery in the shARNT tumor cells retarded the growth of the mice. In addition, in comparisons between the shLacZ and doxycycline-treated shARNT groups, the formation of tumors in the lung was increased in the loss-of-ARNT group (Figure 6D). An increase in the size of the tumors was observed when ARNT was restored in the doxycycline-removed group (Figure 6D). These results suggest that ARNT depletion enhanced tumor metastasis; however, ARNT recovery was essential for tumor growth after the tumor had metastasized to distal organs. The dimerization of ARNT with its partners, such as HIF-1α, Sp1 and AhR, is required for its activity [1, 30]. To further examine the possibility that ARNT partners also participate in ARNT-regulated tumor metastasis, the effect of AhR-knockdown on cell invasion was examined. As shown in Supplementary Figure S9A, invasion was increased in shAhR cells as well as in shARNT cells. Interestingly, the promoter activity of aurora C, which confers tumor growth during tumorigenesis [31, 32], was further increased in cells that co-expressed ARNT and AhR (Supplementary Figure S9B). These results indicated that ARNT may cooperate with AhR to regulate tumor cell growth and metastasis.

### Decreased ARNT expression in late-stage human colorectal cancer

**Figure 7:**
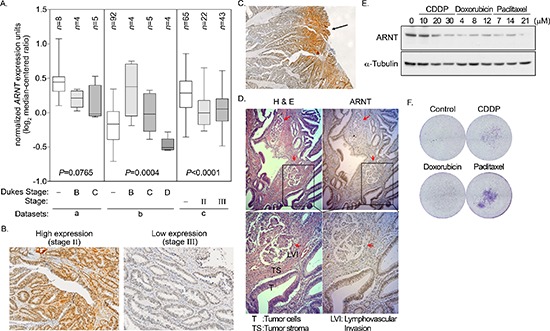
Degradation of ARNT in late-stage colorectal cancer **(A)** Data mining was performed on the cancer microarray database Oncomine 4.0 (Oncomine DB at http://www.oncomine.org). Oncomine box plot of *ARNT* expression levels in normal human colon and colorectal cancer in three datasets from a [53], b [54] and c [55], *respectively*. Values are indicated as the mean ± s.e.m. **(B–D)** Human colorectal cancer specimens were stained with hematoxylin and eosin. High expression and low expression of ARNT was found in the AJCC stage II and stage III colorectal cancers, respectively (×100) **(B)** Superficial colorectal cancers expressed higher levels of ARNT than those in deep sites (×40) **(C)** Colorectal cancer cells invading the lymphatic duct expressed lower levels of ARNT than those in surrounding tumor tissues (×100) **(D) (E)** Cells were treated with cisplatin (CDDP), doxorubicin and paclitaxel for 24 h. Cell lysates were prepared and subjected to SDS-PAGE, then analyzed by Western blotting with antibodies against ARNT and α-Tubulin. **(F)** Cells were treated with cisplatin (CDDP), doxorubicin and paclitaxel for 24 h. The attached/surviving cells were collected by trypsinization and re-seeded for another 3 h. The invasive properties of the residual cells were examined using the invasion assay as described in the “Materials and methods”. The invasive images were examined using a microscope.

To clarify whether ARNT expression was correlated with cancer progression, ARNT expression levels in tissues from different stages of colorectal cancer were examined. First, *ARNT* expression was analyzed using the cancer microarray database from Oncomine 4.0 [33]. *ARNT* expression was significantly down-regulated in late-stage human colorectal cancer (Figure 7A). Analysis of a dataset from GEO [34] that contained 58 colorectal carcinomas also showed decreased *ARNT* expression in late-stage human colorectal cancer (Supplementary Figure S10A). In addition, invasive IPMC had lower ARNT expression levels than non-invasive IPMA (Supplementary Figure S10B). Next, we examined ARNT expression in colorectal cancer specimens using immunohistochemical staining (Figure 7B). The ARNT staining results are summarized in Table 1. ARNT expression in individual tumor specimens was characterized as absent, low or high. Fisher's exact test indicated that ARNT expression was significantly correlated with the pTNM stage, pT status, and pM status (*p* < 0.05 for all comparisons). Kendall's tau (τ)-b correlation analysis revealed that ARNT expression was negatively correlated with the pTNM stage (*p* = 0.002; τ = −0.453), pT status (*p* = 0.007; τ = −0.394), pN status (*p* = 0.002; τ = −0.345), and pM status (*p* = 0.003; τ = −0.480). ARNT expression in the tumor tissues was lower in deep sites compared to superficial sites (Figure 7C). In addition, decreased ARNT expression was observed in lymph-vascular invasion samples compared to neighboring non-metastatic tumor tissue (Figure 7D, red arrow). These results reveal that ARNT expression was decreased in highly invasive and metastatic colorectal cancer cells.

**Table 1:** ARNT IHC intensity is negatively correlated with TNM stage in human colorectal cancer

ARNT expression^*^	No expression (*N* = 15)	Low expression (*N* = 18)	High expression (*N* = 5)		
Characteristic	Number of Patients *n* (%)	Number of Patients *n* (%)	Number of Patients *n* (%)	*P*-value^†^	τ *P*-value^‡^
pTNM stage				0.014	−0.453 0.002
0 or I or II	3 (20.0)	6 (33.3)	4 (80.0)		
III	3 (20.0)	9 (50.0)	1 (20.0)		
IV	9 (60.0)	3 (16.7)	0 (0)		
pT status				0.037	−0.394 0.007
pTis or p T1	0 (0)	2 (11.1)	3 (60.0)		
pT2	1 (6.7)	5 (27.8)	0 (0)		
pT3	12 (80.0)	9 (50.0)	2 (40)		
pT4	2 (13.3)	2 (11.1)	0 (0)		
pN status				0.200	−0.345 0.002
pN0	3 (20.0)	6 (33.3)	4 (80.0)		
pN1	6 (40.0)	8 (44.4)	1 (20.0)		
pN2	6 (40.0)	4 (22.2)	0 (0)		
pM status				0.010	−0.480 0.002
pM0	6 (40)	15 (83.3)	5 (100.0)		
pM1	9 (60.0)	3 (16.7)	0 (0)		
Tumor differentiation				0.148	−0.315 0.060
Well	0 (0)	2 (11.1)	2 (40.0)		
Moderate	14 (93.3)	15 (83.3)	3 (60.0)		
Poor	1 (6.7)	1 (5.6)	0 (0)		

* The IHC results of ARNT were grouped “no expression” (no IHC reactivity or < 5% positive cells/total tumor cells), “low expression” (faint or light brown nuclear staining in at least 5% of total tumor cells), and “high expression” (dark brown nuclear staining in at least 5% of total tumor cells).

† Fisher's exact test

‡ Kendall's tau (τ)-b correlation analysis

**Figure 8:**
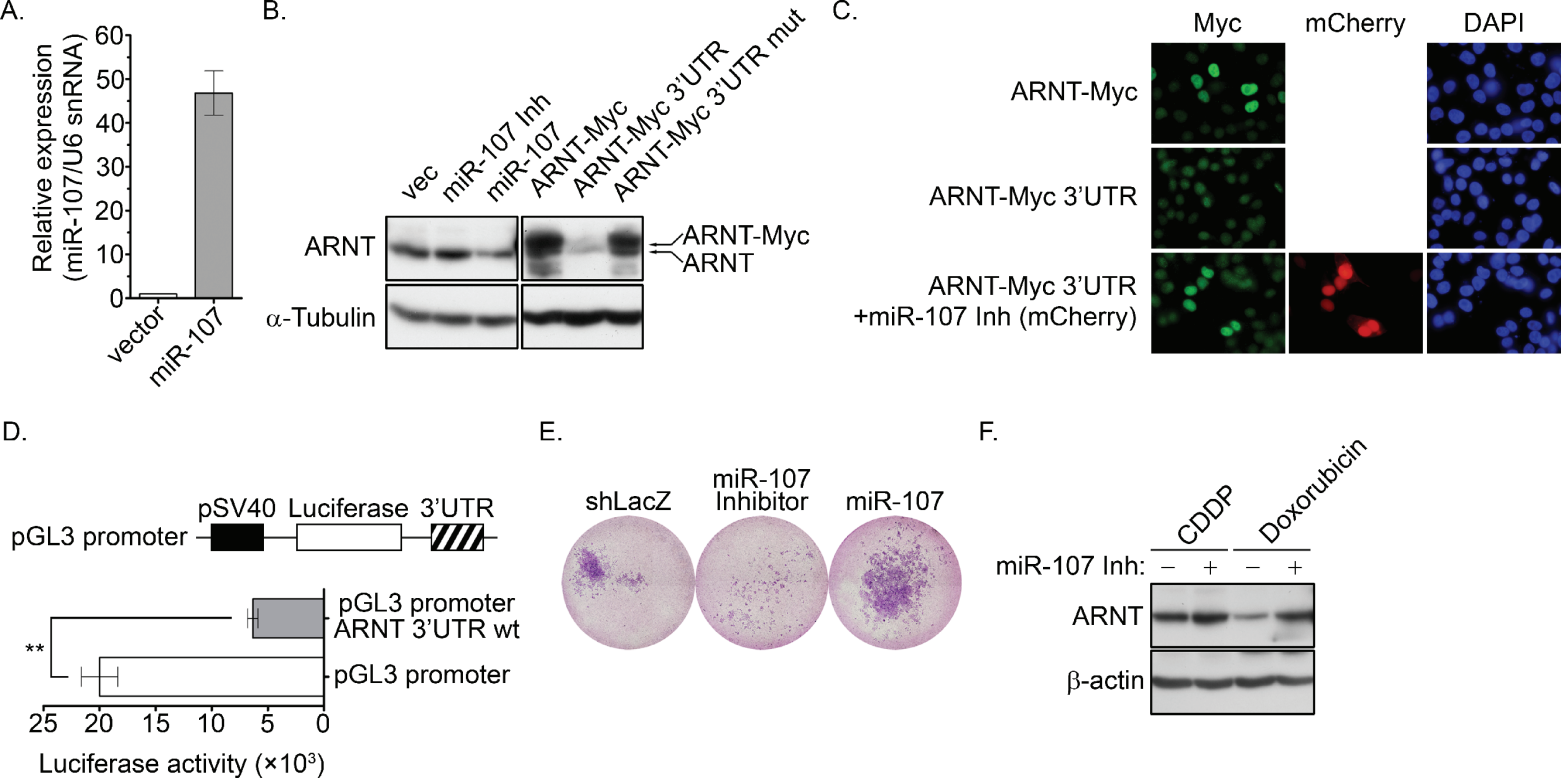
miR-107 targets ARNT to promote cancer cell invasion **(A)** A miR-107 expression construct was transfected into cells. The expression of miR-107 was examined by Q-PCR (GeneCopoeia, Inc., Rockville, MD, USA). **(B)** Cells were transfected with expression vectors including miR-107, miR-107 inhibitor (miR-107 Inh), ARNT-Myc, ARNT-Myc 3′UTR and ARNT-Myc 3′UTR mut via lipofection. The expression of ARNT, ARNT-Myc and α-tubulin was analyzed by Western blotting. **(C)** Cells were transfected with mCherry-labeled miR-107 inhibitor (miR-107 Inh), ARNT-Myc and ARNT-Myc 3′UTR. Anti-Myc antibodies and DAPI were used to stain ARNT and DNA, respectively, in an immunofluorescence analysis. **(D)** ARNT 3′UTR fragments were generated and subcloned into the pGL3-promoter construct, which harbors the SV40 promoter. Cells were transfected with 1 μg of the promoter construct via lipofection. Luciferase activity and protein concentrations were then determined and normalized. Values are indicated as the mean ± s.e.m. **(E)** Cells expressing the miR-107 inhibitor and miR-107 were selected with puromycin. The invasive properties of the cancer cells were examined using the invasion assay. Images for the invasion assay were examined using a microscope. **(F)** Control or miR-107-expressing cells were treated with 20 μM cisplatin (CDDP) and 4 μM doxorubicin for 24 h. Expression of ARNT and α-tubulin were analyzed by Western blotting.

We previously found that ARNT expression was decreased in tumor cells that were treated with cisplatin [4]. Therefore, we hypothesized that tumor metastasis would be induced when ARNT was repressed during chemotherapy. To investigate whether ARNT expression was repressed in cells treated with chemotherapeutic drugs, first-line chemotherapeutic drugs were used to treat tumor cells. As shown in Figure 7E, the expression of ARNT was decreased in cells treated with cisplatin, doxorubicin and paclitaxel. In addition, the residual cells had higher migration ability (Figure 7F). These results indicate that chemotherapy may promote the metastasis of surviving cells via ARNT degradation. Recent reports have shown that overexpression of miR-107 inhibits ARNT expression by targeting the 3′UTR of ARNT [22]. Therefore, we studied whether the expression of ARNT was controlled by miR-107, resulting in enhancement of tumor cell metastasis. As shown in Figure 8A, the expression of miR-107 was elevated when cells were transfected with the miR-107-expressing vector. The expression of miR-107 inhibited ARNT expression, and the inhibition was reversed by a miR-107 inhibitor (Figure 8B). Compared to ARNT-Myc, the expression level of ARNT-Myc with a 3′UTR (ARNT-Myc 3′UTR) was significantly decreased (Figure 8B). The expression level of ARNT-Myc 3′UTR was recovered when the miR-107-targeted sequence was mutated (ARNT-Myc 3′UTR mut). Immunofluorescence staining also demonstrated that the miR-107 inhibitor enhanced the expression of ARNT-Myc 3′UTR (Figure 8C). In addition, the promoter of the ARNT 3′UTR construct exhibited lower activity (Figure 8D). These results indicated that ARNT was a target of miR-107. To further confirm that miR-107-regulated ARNT expression was associated with tumor cell invasion, the invasion assay was performed in miR-107- or miR-107 inhibitor-expressing cells. As shown in Figure 8E, miR-107-expressing cells had higher invasive properties, as did shARNT cells. In contrast, miR-107 inhibitor-expressing cells lost their capacity for invasion (Figure 8E). Interestingly, chemotherapeutic drug-promoted ARNT degradation was reversed by the miR-107 inhibitor (Figures 7E and 8F). These results suggest that the degradation of ARNT in cells treated with chemotherapeutic drugs may be regulated by miR-107.

## DISCUSSION

Our findings show for the first time that ARNT may play both a positive role in tumor growth in either early-stage cancer or in metastatic organs and a negative role in tumor invasion and migration. ARNT depletion promoted the activation of the fibronectin/integrin β1/FAK signaling axis and increased tumor invasion and migration. In addition, ARNT expression was negatively correlated with cancer stage in human colorectal cancer. In contrast, recovery of ARNT in ARNT-deficient metastatic cells increased tumor nodules in the lung. Thus, identifying the stage of cancer progression would have major implications for ARNT-targeted therapies, which are likely more useful in early stages of cancer. However, the challenge of ARNT-targeted therapy is to maintain the expression of nonpathological levels of ARNT in tumors, as this would be expected to prevent metastasis, rather than eliminating ARNT, which could lead to the promotion of metastatic progression.

Coinciding with our finding that ARNT expression plays a dual role in both the enhancement of tumor growth and the inhibition of migration and invasion, several reports have also noted the opposing features of tumor growth and metastasis that are regulated by oncoproteins. For example, stathmin 1 (STMN1) is up-regulated in several types of cancer and is correlated with disease progression and poor prognostic outcome [35]. However, there are contradictory reports that indicate that STMN1 inhibition accelerates the metastatic process [36]. In addition, Sp1 levels strongly increase in the early stages and then decline in the late stages of lung cancer, indicating its dual function as both a metastasis suppressor and oncogene [37]. Our previous studies have shown that ARNT cooperated with Sp1 to regulate the expression of genes that were associated with tumor growth [30]. Activation of AhR, one of the ARNT-associated factors, also induces tumor formation but inhibits cancer cell invasion and metastasis [38–41]. Consistent with these results, we also found that the depletion of AhR enhanced tumor cell invasion. In addition, ARNT and AhR cooperated in stimulating aurora C expression. On the basis of these findings, it appears that the inactivation of ARNT or its partners, such as Sp1 and AhR, inhibits tumor growth; however, it may also promote metastasis, allowing tumor cells to escape from their immediate environment, such as during targeted therapy.

ARNT ablation could be caused by treating cells with chemotherapeutic drugs, as shown in this study. In addition, miR-107 overexpression inhibits ARNT expression by targeting the 3′UTR of ARNT. The expression of miR-107 was also up-regulated in both chemotherapeutic drug-treated colorectal cancer cells or under hypoxic conditions, which is a significant event in the promotion of tumor metastasis [42, 43]. Consistent with our finding that ARNT was decreased in late-stage colorectal cancer, miR-107 is elevated in highly metastatic colorectal cancer tissue [44]. In this study, the expression of the ARNT-Myc 3′UTR in expression vector-transfected cells was less than the expression of ARNT-Myc. We also found that miR-107 overexpression significantly inhibited ARNT expression and promoted tumor cell invasion. However, the miR-107 inhibitor not only reversed the chemotherapeutic drug-induced degradation of ARNT, but inhibited tumor cell invasion. These results indicate that the elevated miR-107 levels found in late-stage cancer and with chemotherapeutic treatment could down-regulate ARNT levels and therefore promote tumor metastasis. Previous reports have revealed that cisplatin-resistant non-small cell lung cancer cells have an increased capacity for inducing metastasis and express several genes involved in tumor development and metastasis [45]. Furthermore, side effects of chemotherapy-induced metastasis have been reported in several studies. Cisplatin and paclitaxel significantly enhanced the expression of VEGFR-1 in endothelial cells and increased its adhesion to tumor cells, therefore enhancing lung metastasis in an experimental mouse model [46]. Cisplatin-induced EMT is correlated with reduced E-cadherin and increased vimentin, Snail, Twist, and matrix metalloproteinase (MMP)-2 expression in ovarian cancer cells [47]. In our study, ARNT depletion due to chemotherapeutic drug treatment enhanced the tumor invasion capabilities of the residual tumor cells. Additionally, ARNT depletion was associated with the expression of fibronectin, N-cadherin, and integrin β1. Whether the degradation of ARNT induced by the drugs is involved in the regulation of the expression of other metastatic genes will be clarified in our next study.

Previously, we and others have shown that ARNT plays an important role in regulating the expression of genes, including COX-2, 12(*S*)-lipoxygenase, p21*^WAF1/CIP1^*, MDR1, VEGF and CYP1A1, in respond to various environmental conditions, such as toxic stress, growth factors and hypoxia [1–4]. In this study, we found that ARNT depletion significantly induced the expression of fibronectin, N-cadherin and integrin β1 and the activation of FAK. This is the first study to reveal that ARNT acts as a suppressor that prohibits EMT progression by reducing metastasis-related gene expression. The mechanisms involved in the ARNT-inhibited gene expression of fibronectin, N-cadherin and integrin β1 remain unknown. In this study, we found that elevated ROS levels and ERK activation in shARNT cells were associated with fibronectin expression and FAK phosphorylation. A recent report also indicates that ROS-induced ERK activation enhances fibronectin expression [28]. These results suggest that the increase in ROS levels at least contributes to ARNT depletion-induced tumor cell metastasis. In addition, recent reports have demonstrated that deferoxamine mesylate (DFO) enhances Ezh2 expression through ARNT induction, which leads to the inhibition of miR-101 expression [48]. In addition, ARNT is essential for HIF-induced miR-210 expression in response to hypoxia in mouse embryonic fibroblasts (MEFs) [49]. These results suggest that ARNT expression induces increased microRNAs levels in cells; therefore, it is possible that ARNT regulates EMT gene expression through the induction of microRNAs. Furthermore, miR-122 overexpression reduces expression of the mesenchymal proteins fibronectin and vimentin, resulting in inhibition of the migration and invasion of hepatocellular carcinoma (HCC) cells [50]. In addition, previous studies have reported that miR-200b and miR-429 repress TGF-β1-induced fibronectin expression in kidney proximal tubular and human primary mesothelial cells [51, 52]. However, further studies should be performed to identify the ARNT-induced microRNAs that could regulate fibronectin.

In conclusion, we provide strong evidence that ARNT depletion enhances tumor migration and invasion through activation of the fibronectin/integrin β1/FAK signaling axis. We demonstrated a clinically relevant correlation between ARNT down-regulation and late-stage colorectal cancer. ARNT may play a positive role in tumor growth (in either early-stage tumor growth or in metastatic organs) but plays a negative role in tumor migration and invasion. Therefore, the efficiency of ARNT-targeted therapy for different stages of cancer should be carefully evaluated.

## SUPPLEMENTAL FIGURES


