# Supplementary material for: Down-regulation of ARNT promotes cancer metastasis by activating the fibronectin/integrin β1/FAK axis
Source: Oncotarget. 2015 Mar 20;6(13):11530–46. doi: 10.18632/oncotarget.3448 (PMC4484474; doi:10.18632/oncotarget.3448)
Supplement: Supplementary file 1 [file oncotarget-06-11530-s001.pdf]

SUPPLEMENTARY FIGURES

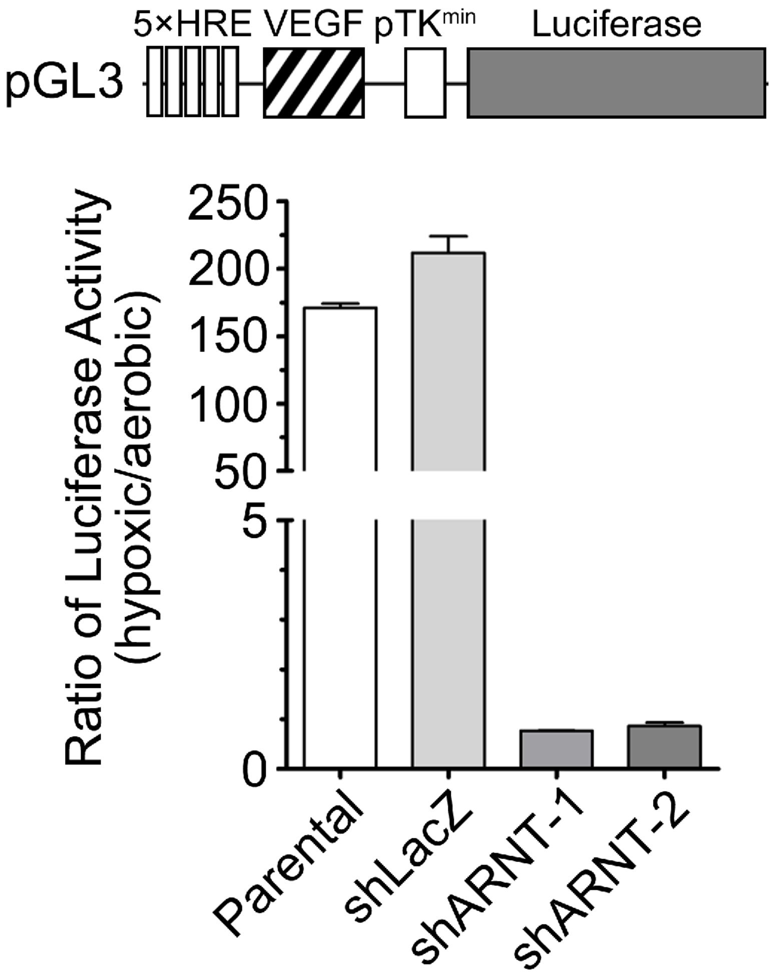

Supplementary Figure S1: Hypoxia-induced luciferase activity is abolished in shARNT cells. The construct containing the pTK promoter with 5 repeats of the hypoxia response element (HRE) and bearing the luciferase gene is presented (upper panel). Cells were transfected with 0.5 µg of plasmid by lipofection and further cultured in 20% oxygen (aerobic) or 5% oxygen (hypoxic) environments overnight. Luciferase activity and protein concentrations were then determined and normalized. The ratio of expression in the hypoxic to aerobic environment is shown. Values represent the mean ± s.e.m. of three determinations.

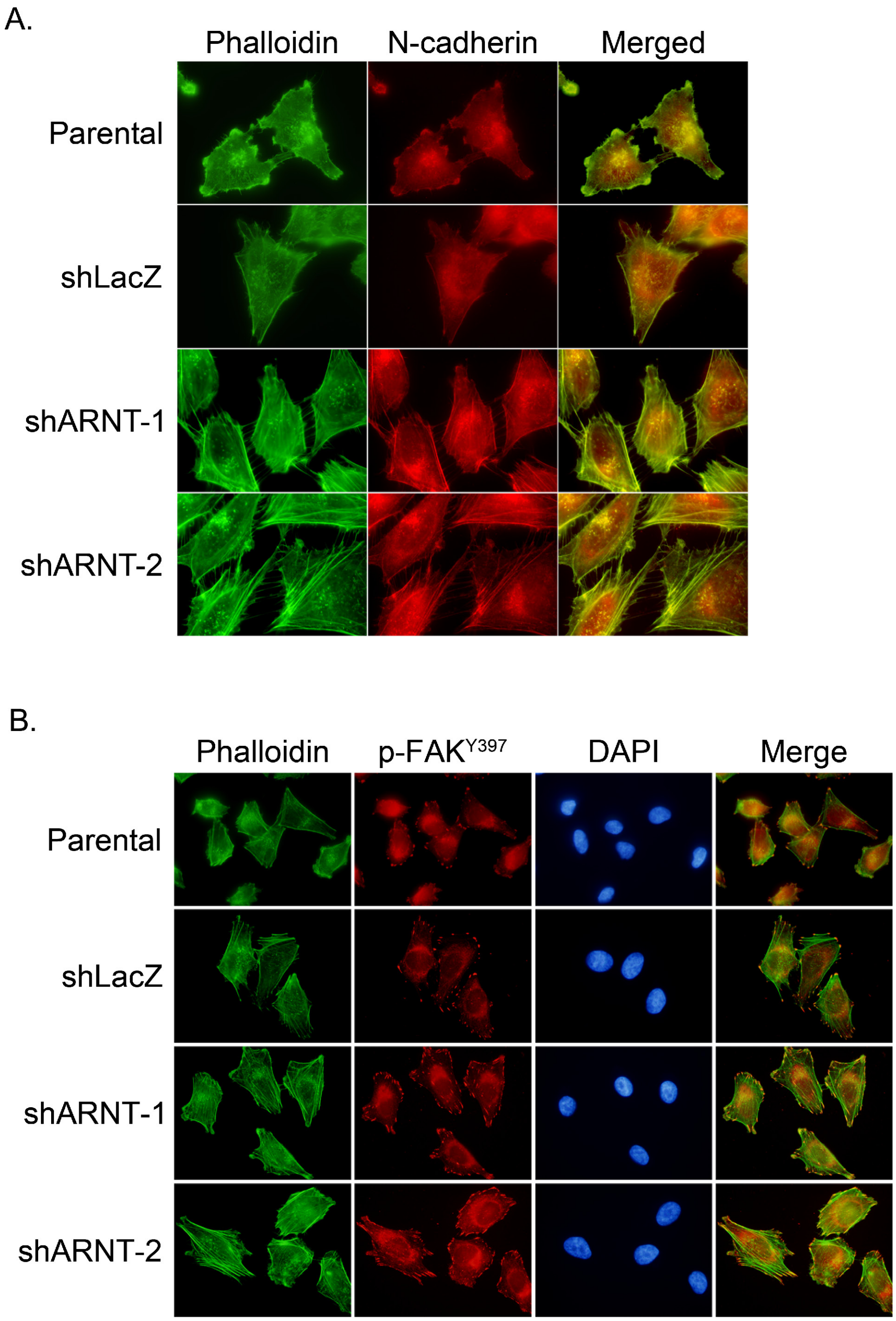

Supplementary Figure S2: Immunofluorescence analysis of F-actin, N-cadherin and the phosphorylation of FAK in cancer cells. (A-B) Paraformaldehyde-fixed cells were permeabilized and probed with Alexa Fluor® 488-conjugated phalloidin (green), Alexa Fluor® 594-conjugated N-cadherin (red) (A) or phosphorylation of FAK^{Y397} **(B)** antibodies. Cells were counterstained with DAPI. Images were taken on a microscope at 600X magnification.

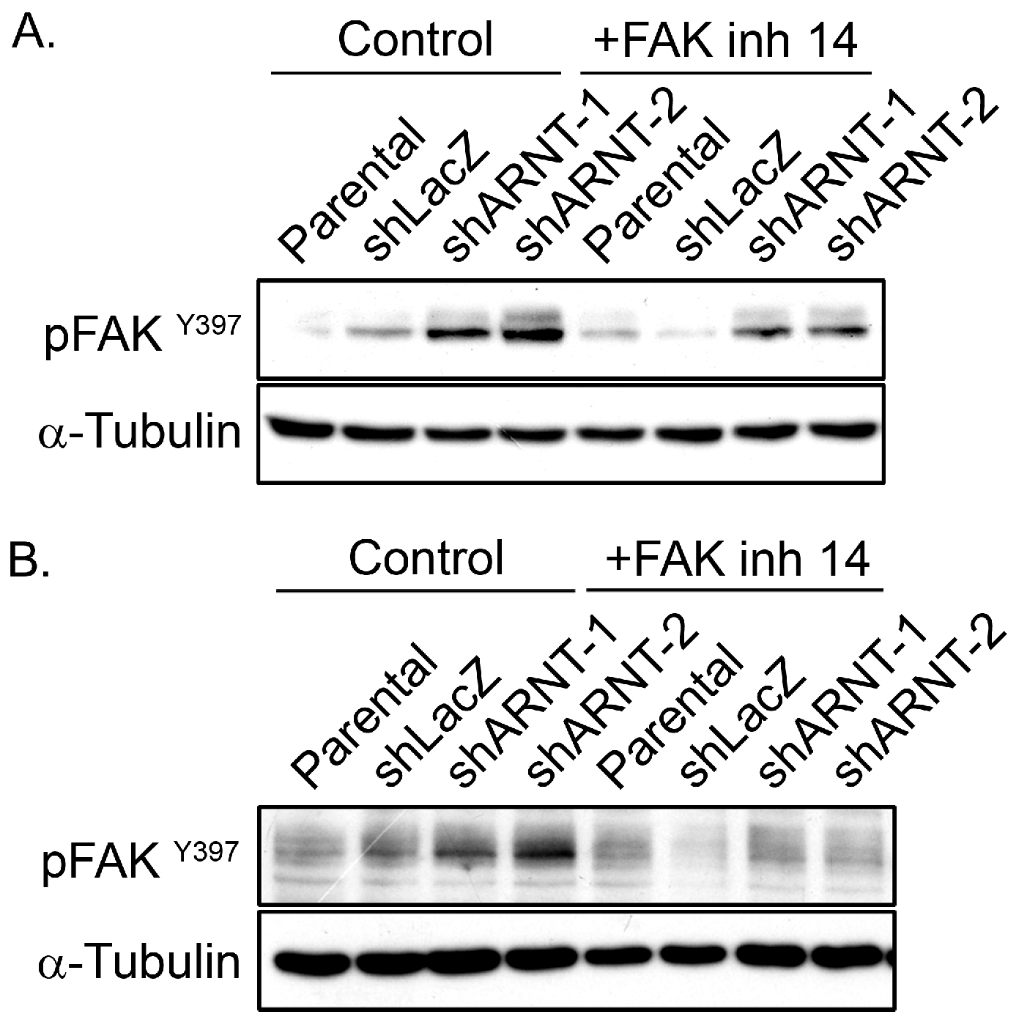

Supplementary Figure S3: FAK inhibitor 14 reduces the phosphorylation of FAK in shARNT cells. **(A-B)** A375 **(A)** and SW480 **(B)** cells were treated with FAK inhibitor 14 at 2 µM and 8 µM, respectively, for 24 h. Cell lysates were prepared and subjected to SDS-PAGE and then analyzed by Western blotting with antibodies against phosphorylation of FAK^{Y397} and α-Tubulin.

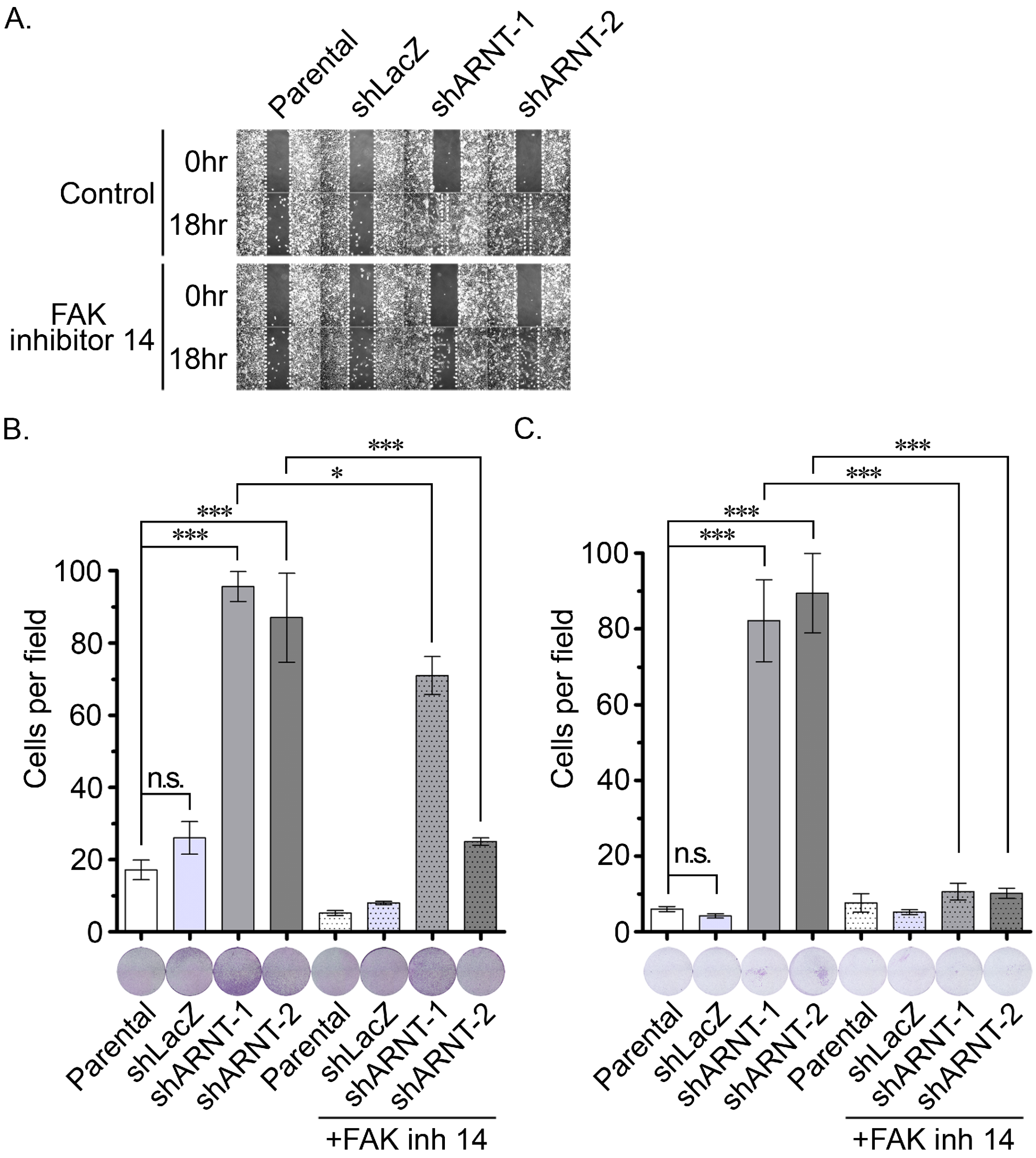

Supplementary Figure S4: Inhibition of FAK activity blocks cell migration and invasion in shARNT cells. **(A)** A375 cells were treated with 2 μM of FAK inhibitor 14 for 18 h. Cell migration was measured by an in-vitro wound-healing assay. **(B-C)** Cells were treated with 8 μM, respectively. FAK inhibitor 14. The migration and invasive properties of cancer cells were examined using migration (B) and invasion (C) assays, respectively, as described in the "Materials and methods". Images for analysis of migration and invasion were examined using a microscope (lower panel). The number of migrating and invasive cells was counted using three randomly chosen fields from three independent experiments (upper panel). Values are indicated as the mean ± s.e.m. *: P<0.05; ***: P<0.001; n.s.: no significant difference.

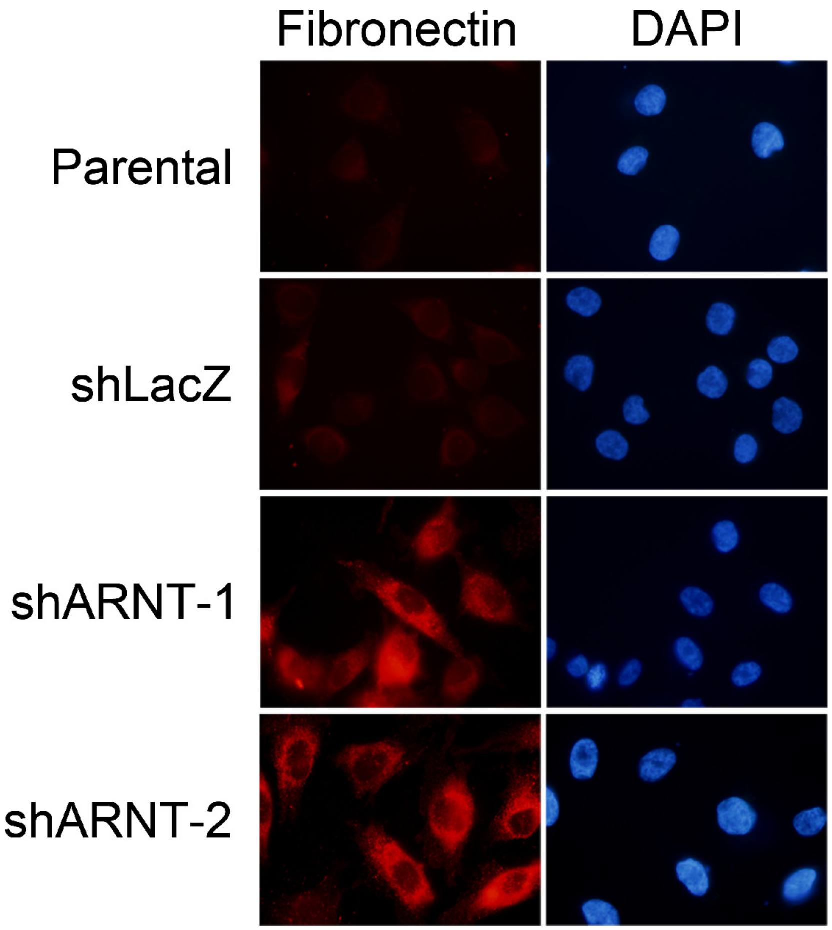

Supplementary Figure S5: Fibronectin is up-regulated in shARNT cells. Paraformaldehyde-fixed cells were permeabilized and probed with fibronectin antibodies. Cells were counterstained with DAPI. Images were taken on a microscope at 600X magnification.

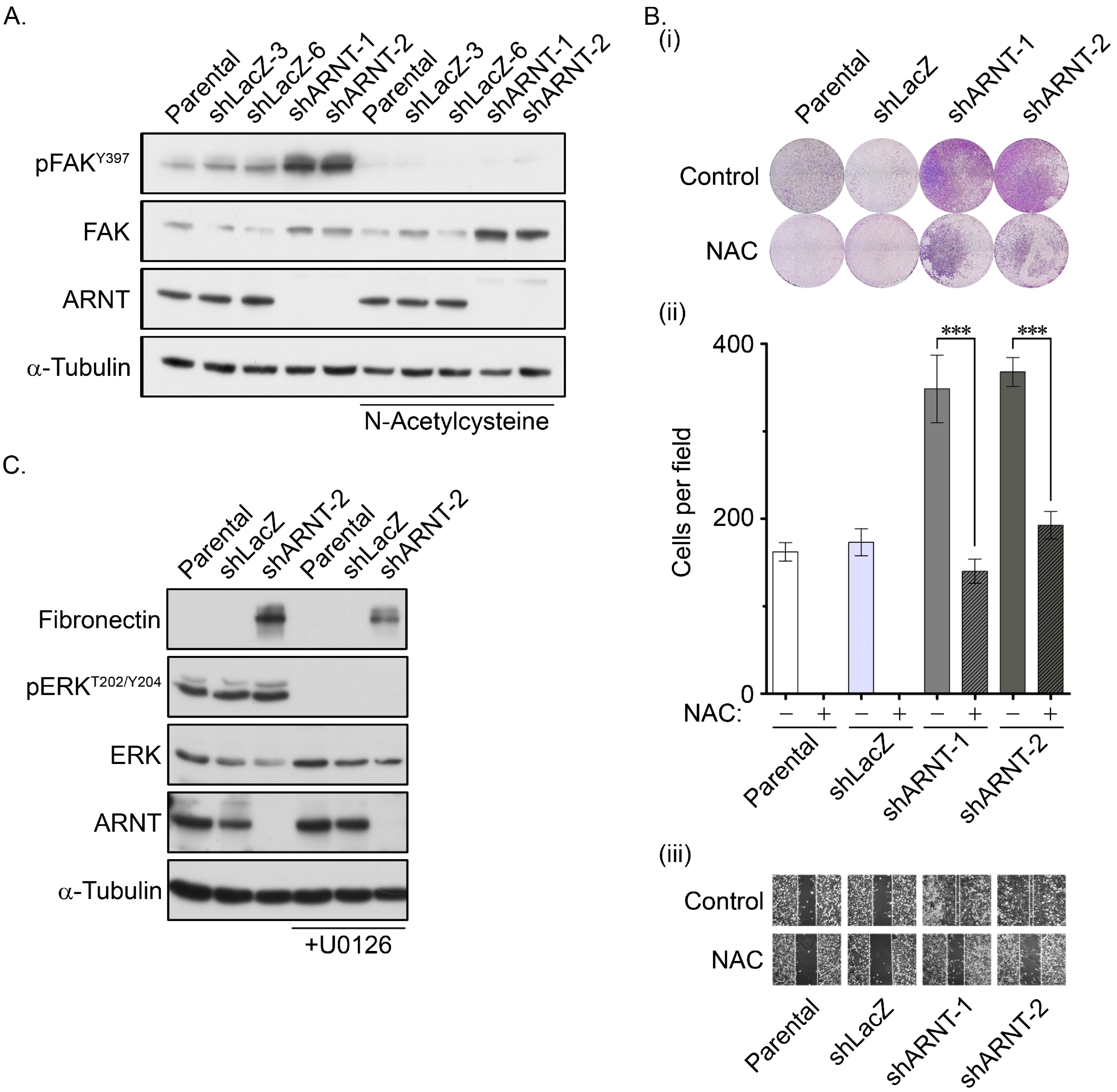

Supplementary Figure S6: Elimination of ROS reduces shARNT cell migration. **(A)** Cells were treated with 10 mM NAC overnight. Cell lysates were prepared and subjected to SDS-PAGE and then analyzed by Western blotting with antibodies against phosphorylation of FAK^{Y397}, FAK, ARNT and α-Tubulin. **(B)** A375 cells were treated with 10 mM NAC for 16h. The migration properties of cancer cells were examined using migration assays (i) as described in the "Materials and methods". The number of migrating cells was counted in five randomly chosen fields under the microscope (ii). Cells were treated with 10 mM NAC for 16 h. Cell migration was measured by an *in-vitro* wound-healing assay (iii). **(C)** Parental, shLacZ and shARNT cells were treated with 10 µM U0126 for 24h. Cell lysates were prepared and subjected to SDS-PAGE and then analyzed by Western blotting with antibodies against fibronectin, phosphorylation of ERK$^{T202/Y204}$, ERK, ARNT and α-Tubulin.

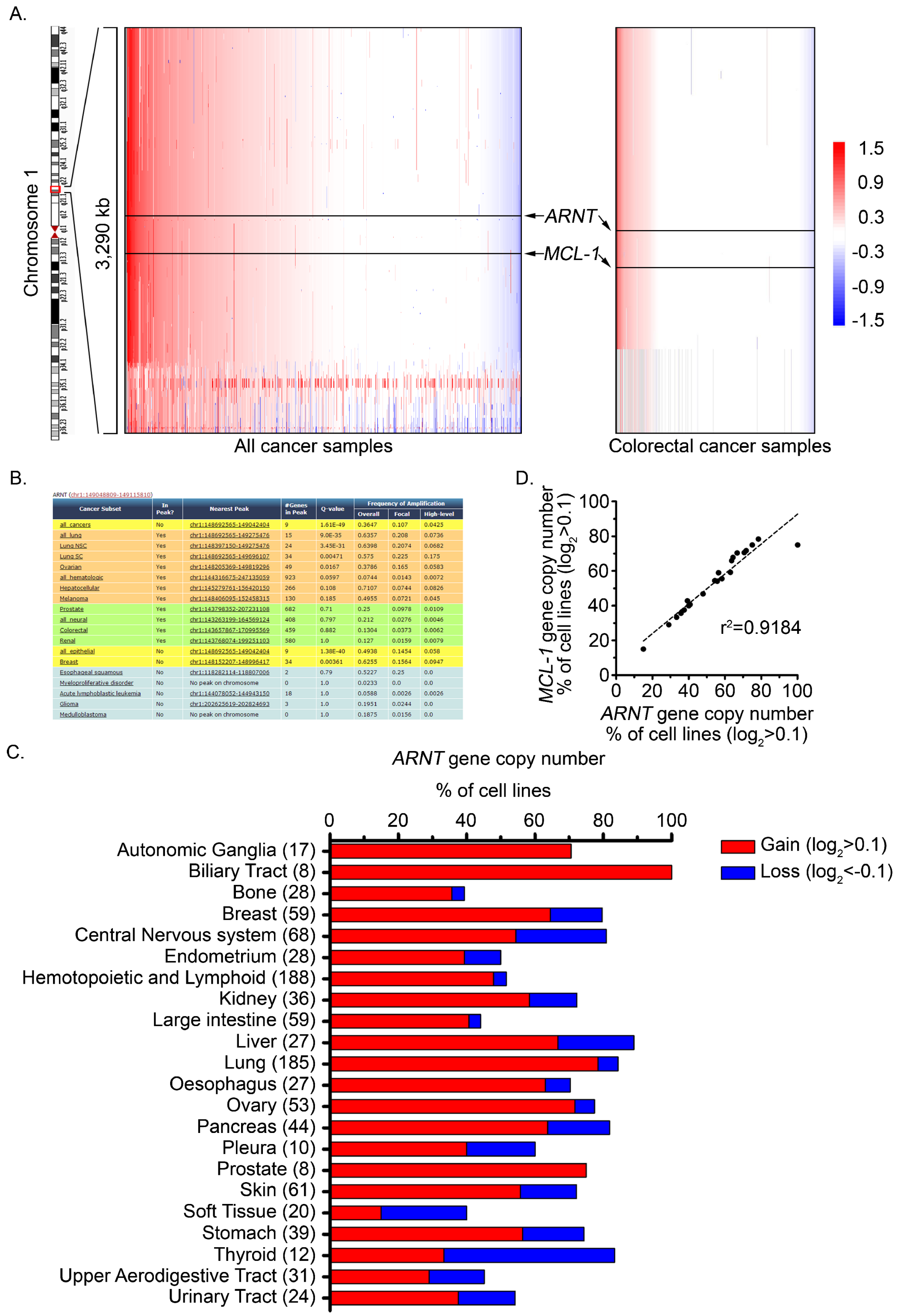

ARNT (chr1:149048809-149115810)

Cancer Subset	In Peak?	Nearest Peak	#Genes in Peak	Q-value	Frequency of Amplification		
					Overall	Focal	High-level
all_cancers	No	chr1:148692565-149042404	9	1.61E-49	0.3647	0.107	0.0425
all_lung	Yes	chr1:148692565-149275476	15	9.0E-35	0.6357	0.208	0.0736
Lung NSC	Yes	chr1:148397150-149275476	24	3.45E-31	0.6398	0.2074	0.0682
Lung SC	Yes	chr1:148692565-149696107	34	0.00471	0.575	0.225	0.175
Ovarian	Yes	chr1:148205369-149819296	49	0.0167	0.3786	0.165	0.0583
all_hematologic	Yes	chr1:144316675-247135059	923	0.0597	0.0744	0.0143	0.0072
Hepatocellular	Yes	chr1:145279761-156420150	266	0.108	0.7107	0.0744	0.0826
Melanoma	Yes	chr1:148406095-152458315	130	0.185	0.4955	0.0721	0.045
Prostate	Yes	chr1:143798352-207231108	682	0.71	0.25	0.0978	0.0109
all_neural	Yes	chr1:143263199-164569124	408	0.797	0.212	0.0276	0.0046
Colorectal	Yes	chr1:143657867-170995569	459	0.882	0.1304	0.0373	0.0062
Renal	Yes	chr1:143768074-199251103	580	1.0	0.127	0.0159	0.0079
all_epithelial	No	chr1:148692565-149042404	9	1.38E-40	0.4938	0.1454	0.058
Breast	No	chr1:148152207-148996417	34	0.00361	0.6255	0.1564	0.0947
Esophageal squamous	No	chr1:118282114-118807006	2	0.79	0.5227	0.25	0.0
Myeloproliferative disorder	No	No peak on chromosome	0	1.0	0.0233	0.0	0.0
Acute lymphoblastic leukemia	No	chr1:144078052-144943150	18	1.0	0.0588	0.0026	0.0026
Glioma	No	chr1:202625619-202824693	3	1.0	0.1951	0.0244	0.0
Medulloblastoma	No	No peak on chromosome	0	1.0	0.1875	0.0156	0.0

Supplementary Figure S7: *ARNT* and *MCL-1* genes are amplified in cancers. **(A)** The copy number of ***ARNT*** and ***MCL-1*** genes were obtained and analyzed from a database (http://www.broadinstitute.org/tumorscape/) of all cancer samples (n = 2,083) and colorectal cancer samples (n = 592). Color bars indicate degree of copy number gain (red) or loss (blue). **(B)** *ARNT* gene copy number was amplified in several types of human cancers collected in The Genome Cancer Atlas (http://cancergenome.nih.gov/) portal. **(C)** *ARNT* gene copy number in the cancer cell lines was obtained from the Cancer Cell Line Encyclopedia (CCLE) [1]. The number in parentheses indicates the sample number that was collected in the database. **(D)** *ARNT* gene copy number was highly correlated with the *MCL-1* gene in the Cancer Cell Line Encyclopedia (CCLE) database.

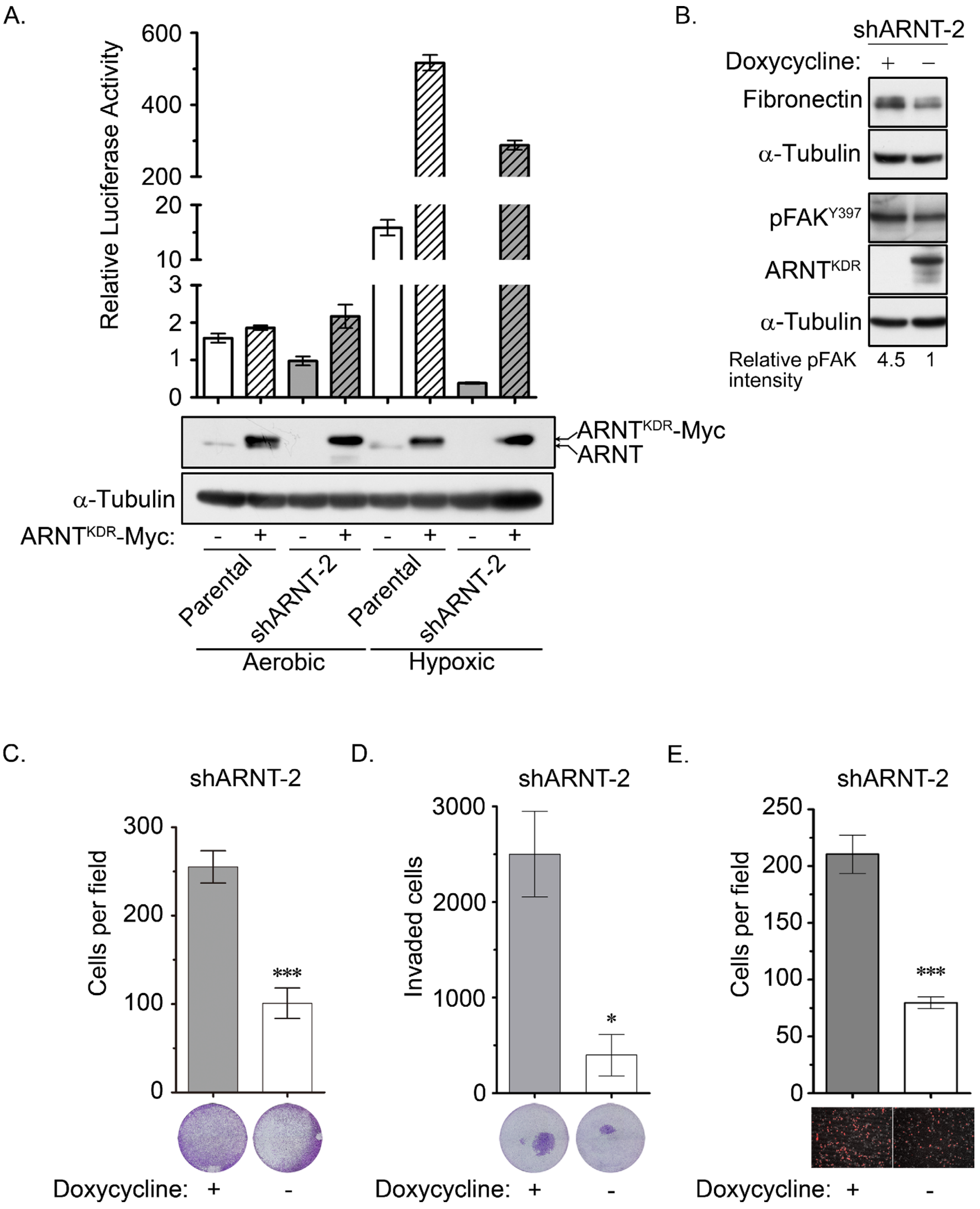

Supplementary Figure S8: ARNT recovery in shARNT cells inhibits cell migration and invasion. **(A)** shARNT cells were transfected with 0.5 µg $ARNT^{KDR}$ Myc-expressing vector via lipofection and further cultured in 20% oxygen (aerobic) or 5% oxygen (hypoxic) environments overnight. Luciferase activity and protein concentrations were then determined and normalized (upper panel). Cell lysates were prepared and subjected to SDS-PAGE and then analyzed by Western blotting with antibodies against ARNT and α-Tubulin (lower panel). Values represent the mean ± s.e.m. of three determinations. **(B)** shARNT cells containing doxycycline-regulated $ARNT^{KDR}$-Myc were selected with puromycin and hygromycin. Cells were maintained with 1 µM doxycycline. Cell lysates were prepared and subjected to SDS-PAGE and then analyzed by Western blotting with antibodies against fibronectin, phosphorylation of FAK^{Y397}, ARNT and α-Tubulin. **(C-E)** The cell migration (C), invasion (D) and cell adhesion (E) assays were performed as described in the "Materials and methods". The migration, invasive and cell adhesion images were examined using a microscope (lower panel). The number of migrating, invasive and adhesive cells was counted using three randomly chosen fields from three independent experiments (upper panel). Values are indicated as the mean ± s.e.m. *: $P< 0.05$; ***: $P< 0.001$.

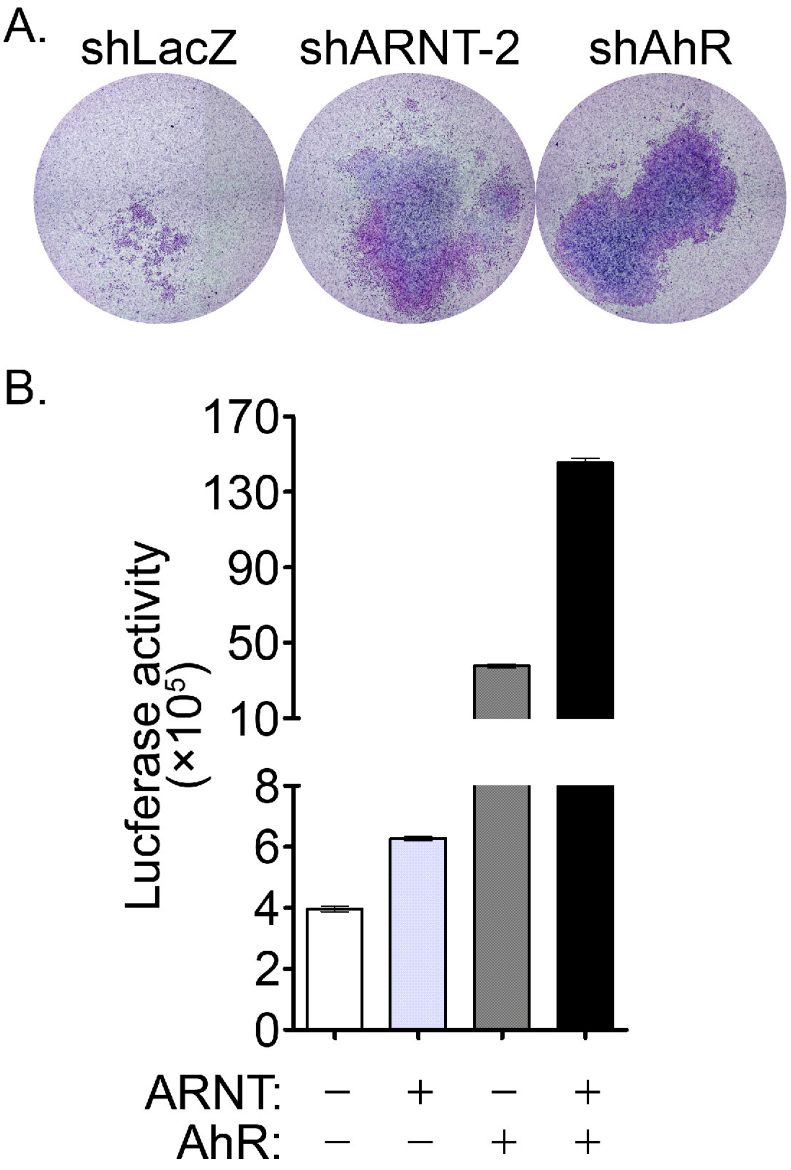

Supplementary Figure S9: The AhR plays the same role as ARNT in the regulation of cell invasion and aurora C kinase expression. (A) The invasive properties of shLacZ, shARNT and shAhR cells were examined using the invasion assay as described in the "Materials and methods". The invasion images were examined using a microscope. **(B)** Cells were transfected with the construct containing the aurora C kinase promoter, ARNT- and AhR- expressing vectors via lipofection for 24 h. The luciferase activity and protein concentration were then determined and normalized. Values are indicated as the mean ± s.e.m.

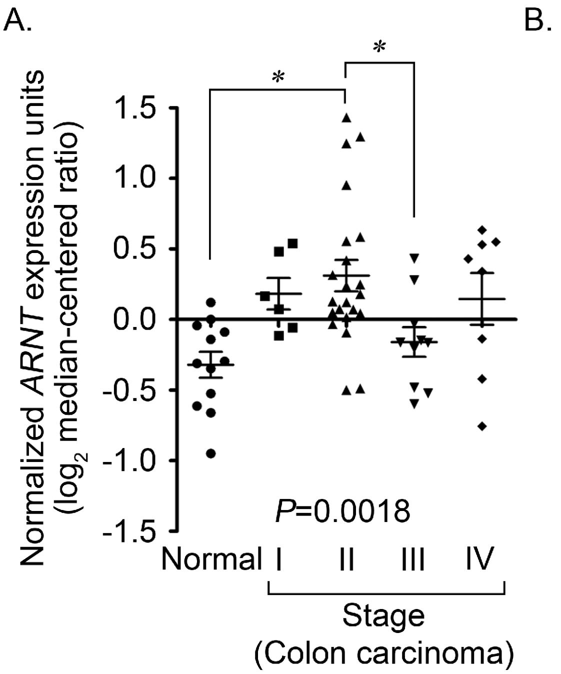

Supplementary Figure S10: Down-regulation of ARNT in cancers. (A) ARNT expression was determined from different stages of human colon carcinoma (GEO data set GSE31905) [2]. **(B)** ARNT expression was classified into three groups in human pancreatic cancer (GEO data set GDS3836) [3]. IPMA: intraductal papillary-mucinous adenoma; IPMC: intraductal papillary-mucinous carcinoma; IPMN: invasive cancer of intraductal papillary-mucinous neoplasm. Values are indicated as the mean ± s.e.m.

REFERENCES

1. Barretina J, Caponigro G, Stransky N, Venkatesan K, Margolin AA, Kim S, Wilson CJ, Lehar J, Kryukov GV, Sonkin D, Reddy A, Liu M, Murray L, Berger MF, Monahan JE, Morais P, et al. The Cancer Cell Line Encyclopedia enables predictive modelling of anticancer drug sensitivity. Nature. 2012; 483:603–307.
2. Anders M, Fehlker M, Wang Q, Wissmann C, Pilarsky C, Kemmner W, Hocker M. Microarray meta-analysis defines global angiogenesis-related gene expression signatures in human carcinomas. Mol Carcinog. 2013; 52:29–38.
3. Hiraoka N, Yamazaki-Itoh R, Ino Y, Mizuguchi Y, Yamada T, Hirohashi S, Kanai Y. CXCL17 and ICAM2 are associated with a potential anti-tumor immune response in early intraepithelial stages of human pancreatic carcino-genesis. Gastroenterology. 2011; 140:310–321.
